# Histidine Residues Are Responsible for Bidirectional Effects of Zinc on Acid-Sensing Ion Channel 1a/3 Heteromeric Channels

**DOI:** 10.3390/biom10091264

**Published:** 2020-09-02

**Authors:** Qian Jiang, Andrew M. Peterson, Yuyang Chu, Xiaolan Yao, Xiang-ming Zha, Xiang-Ping Chu

**Affiliations:** 1 Department of Biomedical Sciences, School of Medicine, University of Missouri-Kansas City, Kansas City, MO 64108, USA; jiangqi@umkc.edu (Q.J.); amp8m6@mail.umkc.edu (A.M.P.); 2 Feinberg School of Medicine, Northwestern University, Chicago, IL 60611, USA; yuyang.chu@northwestern.edu; 3 Division of Molecular Biology and Biochemistry, School of Biological and Chemical Sciences, University of Missouri-Kansas City, Kansas City, MO 64110, USA; yaoxia@umkc.edu; 4 Department of Physiology and Cell Biology, College of Medicine, University of South Alabama, Mobile, AL 36688, USA; zha@southalabama.edu

**Keywords:** acid-sensing ion channels, ASIC1a, ASIC3, zinc, mutation, histidine, modulation

## Abstract

Acid-sensing ion channel (ASIC) subunits 1a and 3 are highly expressed in central and peripheral sensory neurons, respectively. Endogenous biomolecule zinc plays a critical role in physiological and pathophysiological conditions. Here, we found that currents recorded from heterologously expressed ASIC1a/3 channels using the whole-cell patch-clamp technique were regulated by zinc with dual effects. Co-application of zinc dose-dependently potentiated both peak amplitude and the sustained component of heteromeric ASIC1a/3 currents; pretreatment with zinc between 3 to 100 µM exerted the same potentiation as co-application. However, pretreatment with zinc induced a significant inhibition of heteromeric ASIC1a/3 channels when zinc concentrations were over 250 µM. The potentiation of heteromeric ASIC1a/3 channels by zinc was pH dependent, as zinc shifted the pH dependence of ASIC1a/3 currents from a pH_50_ of 6.54 to 6.77; whereas the inhibition of ASIC1a/3 currents by zinc was also pH dependent. Furthermore, we systematically mutated histidine residues in the extracellular domain of ASIC1a or ASIC3 and found that histidine residues 72 and 73 in both ASIC1a and ASIC3, and histidine residue 83 in the ASIC3 were responsible for bidirectional effects on heteromeric ASIC1a/3 channels by zinc. These findings suggest that histidine residues in the extracellular domain of heteromeric ASIC1a/3 channels are critical for zinc-mediated effects.

## 1. Introduction

Recently, protons have been identified as neurotransmitters in the brain [1]. One of the candidates for sensing protons is acid-sensing ion channels (ASICs) [2,3]. ASICs are activated by a drop in pH from the physiological level of 7.4 to pathological levels (7.2 and below) and three subunits of ASICs form a functional channel [4,5]. Therefore, activation of ASICs most likely contributes to the pathophysiological process during disease state [6,7,8]. ASICs belong to the degenerin/epithelial Na^+^ channel superfamily [9,10], and are highly expressed in both central and peripheral sensory neurons [11,12,13,14]. To date, four genes (*ASIC1*–*ASIC4*), encoding at least six ASIC subunits, have been cloned [2]. Each ASIC subunit has two transmembrane domains, a large ectodomain with rich histidine residues, and short cytoplasmic N- and C-termini [15,16,17]. The acid-sensing ion channel 1a (ASIC1a) has distinct properties as compared with ASIC3 [15]. The following properties distinguish ASIC1a from ASIC3: (1) although they is an overlap between each other, ASIC1a is dominantly expressed in the brain [2], whereas the expression of ASIC3 in the nervous system is mostly limited to peripheral sensory neurons such as the dorsal root ganglion (DRG) [18,19]; (2) homomeric ASIC1a channels are permeable to Ca^2+^, whereas homomeric ASIC3 channels have no Ca^2+^ permeability [20,21]; (3) the half-maximal activation of pH (pH_50_) of ASIC1a is approximately at 6.2 by a drop in pH from 7.4 [2,20], whereas the pH_50_ for ASIC3 is about 6.8 [22]; (4) pharmacologically, homotrimeric ASIC1a is inhibited by spider toxin PcTx1 with a half maximal inhibitory concentration (IC_50_) of 1 nM and homotrimeric ASIC3 is blocked by sea anemone APETx2 with an IC_50_ of 63 nM [23,24,25,26,27]; (5) functionally, ASIC1a channels mostly contribute to neurological and psychological diseases due to their calcium permeability, whereas ASIC3 channels are mainly involved in pain modulation [28,29,30,31,32].

Although ASIC1a and ASIC3 subunits are expressed in non-neuronal tissue at low levels [11], they are dominantly expressed in the nervous system. ASIC1a is a critical member of the ASICs family and mainly expressed in the brain [11,16]. During the physiological process, ASIC1a is involved in synaptic transmission [1,33], learning and memory [11,34], fear conditioning [35], and pain modulation [36,37]. For example, ASIC1a channels contribute to both long-term potentiation (LTP) and long-term depression [11,34]. During the pathological process, ASIC1a channels contribute to neurological and psychological diseases such as brain ischemia [20,38], seizure [39], traumatic brain injury [40], spinal cord injury [41], Parkinson’s disease [42], Huntington’s disease [43], axonal degeneration in experimental autoimmune encephalomyelitis [44], and cocaine addiction [45,46]. Thus, targeting ASIC1a becomes increasingly important to combat these diseases [20,30].

ASIC3 has been primarily found in peripheral sensory neurons and has been involved in pain modulation [28,32,47]. For example, ASICs in DRG neurons contributed to opioid-induced hyperalgesia by differential modulation of ASIC1a and ASIC3, respectively [48]. Other studies have shown that ASIC3 and ATP-sensitive P2X receptors have tightly associated with each other to form a protein complex for pain modulation [49]. Moreover, recent studies have shown that ASIC3 could also exist in the brain. For example, genetic deletion of the ASIC3 gene increased dopamine release in the dorsal striatum, impaired LTP in corticostriatal circuits, and enhanced self-grooming behavior in mice [50]. Thus, ASIC3 channels are also critical biomolecules for drug targeting [25,27].

ASICs can function as homomeric and heteromeric channels [15]. While many studies have examined ASIC1a/2 and ASIC3/2 heterotrimers, we know relatively little about ASIC1a/3 heterotrimers. Although ASIC1a is mainly present in the brain, whereas ASIC3 is primarily in the periphery, several studies have reported the co-existence of both ASIC1a and ASIC3 in the same cell types. For example, ASICs in mouse skeletal muscle afferents are heterotrimers composed of ASIC1a, ASIC2, and ASIC3 subunits [51]. Electrophysiological studies from ASIC knock-out mouse suggested that ASICs in muscle afferents could be a combination of ASIC1a/3 and ASIC2a/3 channels [51]. Recently, another study showed that ASIC1a/2/3 or ASIC1a/3 heterotrimers could be involved in muscle pain [52]. These studies suggest that, to better interpret how ASICs contribute to physiology in these cells, it is important to characterize the properties of ASIC1a/3 heterotrimers. 

Due to the important roles that ASIC1a and ASIC3 play in the nervous system, regulation or modulation of heteromeric ASIC1a/3 channels are critical to pharmacological agents and endogenous small molecules during physiological and pathological conditions [8,31]. For example, recent studies have shown that homomeric ASIC1a was modulated by the alkaloid daurisoline isolated from the Chinese herb *Menispermum dauricum* [53]. ASIC3 channels were also modulated by endogenous isoquinoline alkaloids through reverse steady-state desensitization [54].

ASIC1a and ASIC3 channels are regulated by physiological concentrations of zinc [55,56]. Our previous studies have shown that high-affinity zinc inhibited homomeric ASIC1a channels with an IC_50_ of approximately 10 nM [57]. Pretreatment, but not co-application with zinc, during pH drop, inhibited homomeric ASIC3 channels in a sharp range of zinc concentrations with an IC_50_ of 61 µM [22,58]. The effects of zinc on ASICs could have important pathophysiological implications because zinc plays a critical role in the pathogenesis of several neurological and psychological diseases, such as stroke, traumatic brain injury, epilepsy, Alzheimer’s disease, Parkinson’s disease, and alcohol abuse [59,60,61,62,63]. 

Although electrophysiological and pharmacological studies have shown that ASIC1a and ASIC3 subunits function as homomeric or heteromeric channels [64], the physiological role and pharmacological profile of heteromeric ASIC1a/3 channels are limited. For example, heteromeric ASIC1a/3 channels are modulated by cadmium, anions, neuropeptide, and PcTx1 [65,66,67,68]. The regulation or modulation of heteromeric ASIC1a/3 by zinc remains uncertain, even given the high expression of ASIC1a and ASIC3 subunits in the brain and peripheral sensory neurons, respectively. Whether or not the heteromeric ASIC1a/3 channels contribute to neurological and psychological diseases is also unclear. Here, we performed a detailed analysis of zinc regulation and modulation of heteromeric ASIC1a/3 channels and found that zinc exerted dual effects on heteromeric ASIC1a/3 currents. Furthermore, using a combination of immunostaining, Western blot, whole-cell patch-clamp recording, amino acid modifiers, and histidine mutants of ASIC1a and ASIC3 expressed in Chinese hamster ovary (CHO) cells, we found that histidine residues 72 and 73, and histidine 83 in the extracellular domain of the heteromeric ASIC1a/3 were responsible for the identified effects.

## 2. Materials and Methods

### 2.1. Transient Expression of ASICs in Chinese Hamster Ovary (CHO) Cells

**Figure 1:**
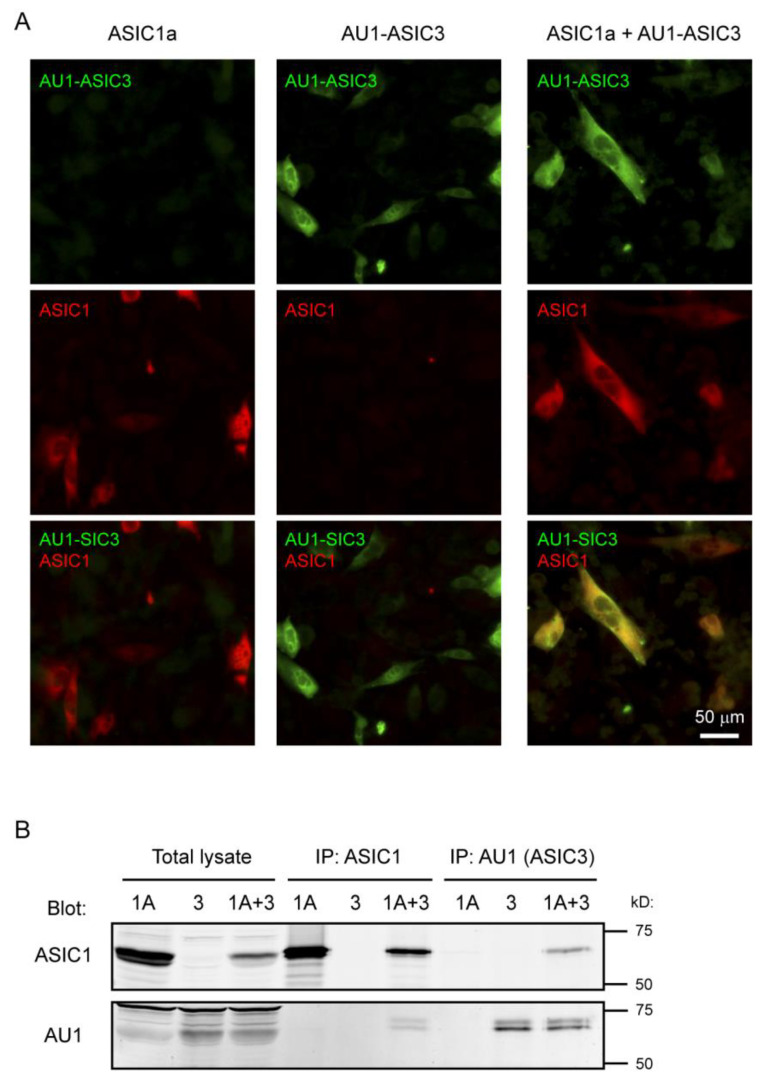
Co-expressed acid-sensing ion channel subunits 1a and 3 (ASIC1a and ASIC3) formed heteromeric complex in Chinese hamster ovary (CHO) cells. (**A**) Immunostaining shows ASIC1 and ASIC3 expression in transfected cells. CHO cells were transfected with ASIC1a, AU1-ASIC3, or ASIC1a + AU1-ASIC3 (1:2 ratio). CHO cells were stained for AU1-ASIC3 (green) and ASIC1 (red) as described in the Materials and Methods section. Note that, when co-transfected into the CHO cell (right panel), most of the ASIC1a expressing cells also were positive for AU1-ASIC3 staining; (**B**) ASIC1a and ASIC3 co-immunoprecipitate from transfected cells. CHO cells were transfected with ASIC1a, AU1-ASIC3, or ASIC1a + AU1-ASIC3 (1:2 ratio). Immunoprecipitation was performed using a goat anti-ASIC1 or goat anti-AU1-ASIC3. Total lysate and immunoprecipitation (IP) fractions were blotted for ASIC1 (rabbit) or AU1-ASIC3 (mouse) as indicated.

The methods for tissue culture of CHO cells (American Type Culture Collection, Manassas, VA, USA) and protocol of transfection of CHO cells with various ASIC subunits have been reported in detail previously [22,57]. Briefly, CHO cells were cultured in standard F12 medium (American Type Culture Collection, Manassas, VA, USA) supplemented with 10% fetal bovine serum at 37 °C in a CO_2_ incubator. The CHO cells were digested with trypsin-EDTA and plated on a 35 mm culture dish at 10 to 15% confluence, and then incubated to recover for 24 h, at 37 °C. At ~50 to 70% confluence, CHO cells were transiently transfected with expression vectors containing rat ASIC1a (gene ID: 79123), rat ASIC3 (gene ID: 286920), both cDNA or histidine mutants, and enhanced green fluorescent protein (eGFP) at a 1:0.25 molar ratio (Invitrogen, San Diego, CA) using X-tremeGENE HP transfection reagent (Roche Diagnostics, Indianapolis, IN, USA). For co-overexpression of ASIC1a and ASIC3, we used a 1:2 ratio and a 2:1 ratio, as shown in Figure 1. For co-overexpression of ASIC1a or its histidine mutants with ASIC3 or its histidine mutants, the 1:2 ratio of ASIC1a/ASIC3 was used. Cultures with transfection of ASICs were used for electrophysiological recording 48 to 72 h after transfection. The cDNA of the rat ASIC1a and rat ASIC3 clone were a gift from Drs. R. Waldmann and M. Lazdunski (Institut de Pharmacologie Moleculaire et Cellulaire, Centre National de Scientifique, Valbonne, France).

### 2.2. Immunostaining and Imaging of ASIC1a and ASIC3 in CHO Cells

CHO cells were plated at a density of 2000 cells per well in the 8-well chambered coverglass, which had been precoated with collagen. Transfection was performed on the second day. Two days after transfection, cells were fixed with 4% paraformaldehyde for 10 min. Immunofluorescence was performed similar to previous studies [69]. For ASIC1, we used a rabbit ASIC1 (DKG) antibody (1:500 dilution), which had been characterized previously [70]. Since ASIC3 antibodies were typically not robust, we used an expression construct which encodes an AU1-ASIC3 fusion gene, and stained ASIC3 using a mouse AU1-ASIC3 antibody (MMS-130P, 1:500 dilution, Biolegends, San Diego, CA, USA). Primary antibodies were incubated at 4 °C overnight, washed 3 times with antibody dilution buffer (PBS, 2% horse serum, 1 mg/mL BSA, 0.05% Triton X-100), incubated with Alexa 488-conjugated goat anti-mouse (1:500) and Alexa 568-conjugated donkey anti-rabbit (1:500) antibodies, for 1 h, at room temperature. Following 3 washes with antibody dilution buffer, cells were imaged using an Olympus IX70 inverted epifluorescence microscope with band filters. Images were captured with a 20× lens with a SPOT cooled CCD camera and SPOT imaging software.

### 2.3. Co-Immunoprecipitation and Western Blot

For co-immunoprecipitation (co-IP), cells were transected in 10 cm dishes. At 24–48 h after transfection, cells were washed 2 times with ice-cold PBS, then removed by scraping into 700 µl of lysis buffer (PBS, 1% Triton X-100, with freshly added protease inhibitor cocktail (Roche Diagnostics, Indianapolis, IN, USA). Lysate was incubated on ice for 20 min, cleared by centrifugation for 15 min at 21 kg, at 4 °C. The supernatant was precleared with 60 µL of Protein G agarose beads and 300 µL of cleared lysate was used per IP. Standard immunoprecipitation with 2 µg anti-ASIC1 (goat IgG, SC-13905, Santa Cruz, CA, USA) or anti-AU1-ASIC3 (goat IgG, PA1-26547, ThermoFisher, ‎Waltham, MA, USA) antibody was performed, for 2 h, at 4 °C. The complex was captured with 60 µl of immobilized Protein G agarose beads, washed 2 times with ice-cold PBS 1% triton, and 2 times with PBS. Precipitated proteins were eluted by adding 60 µL 2× SDS sample buffer. 

For Western blot analysis, total lysates were sonicated briefly and cleared by centrifugation. Before loading, 1/2 volume of 3× SDS sample buffer was added to the lysates, and incubated at 50 °C for 20 min. The samples were separated by 10% SDS-PAGE and transferred to nitrocellulose membranes. Blotting was performed according to instructions of the Odyssey Imaging System (Li-cor, Lincoln, NE, USA). Briefly, membranes were blocked in blocking buffer (0.1% casein in 0.2× PBS pH 7.4) for 1 h. Primary antibodies (rabbit anti-ASIC1 and mouse anti-AU1-ASIC3, both at 1:500 dilution) were diluted with blocking buffer containing 0.1% Tween-20 and incubated at 4 °C overnight or at room temperature, for 2 h. Secondary antibodies were diluted in blocking buffer containing 0.1% Tween-20 and 0.01% SDS and incubated at room temperature for 1 h. Blots were imaged using an Odyssey Infrared Imaging System according to manufacturer’s instructions.

### 2.4. Whole-Cell Patch-Clamp Recording

Whole-cell patch-clamp recording was performed, as described previously [71,72,73]. Patch electrodes, whose resistance ranged from 3 to 6 MΩ when filled with intracellular solution, were constructed from thin-walled borosilicated glass (1.5 mm diameter, WPI, Sarasota, FL, USA) on a two-stage puller (PC-10, Narishige, Tokyo, Japan). Whole-cell currents were triggered by a drop in pH from 7.4 to various levels (e.g., 6.8) at a holding potential of −60 mV and recorded using Axopatch 200B amplifiers (Axon CNS, Molecular Devices, Foster City, CA, USA). Data were filtered at 2 kHz and digitized at 5 Hz using Digidata 1440 DAC units (Axon CNS, Molecular Devices, Foster City, CA, USA). The on-line acquisition was done using pCLAMP software (Version 10.2, Axon CNS, Molecular Devices, Foster City, CA, USA).

In general, ASIC channels were activated by a drop in pH from 7.4 to specific target levels every 2 min to allow for a complete recovery of the channel from desensitization. During each experiment, a voltage step of –10 mV from the holding potential (−60 mV unless specified otherwise) was applied periodically to monitor the cell capacitance and the access resistance. Recordings in which either the access resistance or the capacitance changed by more than 10% during the experiment were excluded from data analysis.

### 2.5. Site-Directed Mutagenesis

The site-directed mutagenesis was performed, as described previously [71,72,73]. Briefly, ASIC1a and ASIC3 point mutations were made using the Quick-Change Site-Directed Mutagenesis system (Stratagene, La Jolla, CA, USA) in accordance with the manufacturer’s protocol. The primers were obtained from Sigma-Genosys (The Woodlands, TX, USA). Mutations were confirmed by restriction enzyme digest and DNA sequence analysis. In all cases, the entire rat ASIC1a and rat ASIC3 cDNAs were sequenced to determine if any nonspecific mutations were introduced. The sequence analyses of ASIC1a WT, ASIC3 WT, and all mutants are listed in Supplementary Materials.

### 2.6. Solutions and Compounds

Standard extracellular fluid (ECF) contained (mM) 140 NaCl, 5.4 KCl, 2.0 CaCl_2_, 1.0 MgCl_2_, 20 HEPES, and 10 glucose (pH 7.4, 320~330 mOsm). For solutions with pH of 6.0 or lower, MES was used instead of HEPES for more reliable pH buffering [71,72,73]. The pipette solution contained (mM) 140 K Gluconate, 10 HEPES, 11 EGTA, 2 TEA, 1 CaCl_2_, 2 MgCl_2_, and 4 K_2_ATP (pH 7.2~7.3, 290~300 mOsm). PcTx1 and APETx2 were purchased from Alomore Labs (Jerusalem, Israel). Other chemicals were purchased from Sigma-Aldrich (St. Louis, MO, USA). A multibarrel perfusion system (SF-77, Warner Instrument Co., Hamden, CT, USA) was employed to achieve a rapid exchange of extracellular solutions. For the pretreatment with zinc protocol, zinc was present in the ECF at both pH 7.4 and lower pH (e.g., 6.8); for the co-application protocol, zinc was only present in the ECF at a lower pH. NaOH was used to adjust the pH of ECF with zinc. The histidine-modifying reagent, diethylpyrocarbonate (DEPC), was dissolved in ECF to 0.6 mM concentration. This method was reported in our previous publications [68,69].

### 2.7. Data Analysis

All data were analyzed using Clampfit 10.2 software (Axon CNS, Molecular Devices, Foster City, CA, USA). For half-maximum excitatory (EC_50_) and inhibitory concentration (IC_50_) curves of zinc, a pH of 6.8 triggered ASIC1a/3 currents; without zinc treatment was used as a control. For treatment with different concentrations of zinc (1, 3, 10, 30, 100, 300, and 1000 µM), ASIC1a/3 currents were normalized to control values without zinc treatment. Normalized values were fitted to the Hill equation to obtain EC_50_ and IC_50_ values, respectively.

For pH activation curves, the ECF flowing out of one barrel of the perfusion system was pH 7.4, whereas the ECF flowing out of the second barrel was switched to pH 7.0, 6.8, 6.5, 6.0, 5.0, and 4.0 sequentially using the SF-77B fast perfusion system (Warner Instrument Co., CT, USA). Acid-triggered currents at each pH were normalized to the peak current activated at pH 5.0. Normalized values were fitted to the Hill equation using SigmaPlot 10 software to obtain pH_50_ values (half value of the maximum pH).

To determine the sustained component of the heteromeric ASIC1a/3 currents. The values were measured at the time point of 6 s, after a pH drop.

### 2.8. Statistics

Statistical analyses were carried out using SigmaPlot software. Significant differences between mean values from each experimental group were tested using a Student’s t-test for two groups and one-way analysis of variance (ANOVA) for all pair-wise multiple comparisons (Bonferroni method). Differences were considered to be significant if *p* < 0.05.

## 3. Results

### 3.1. Co-Expressed ASIC1a and ASIC3 Formed Hetermomeric Complex in CHO Cells

To study ASIC1a/3 heteromeric channels, we co-transfected expression constructs encoding ASIC1a and ASIC3 into CHO cells. To determine whether or not ASIC1a and ASIC3 were co-expressed in the same transfected cell, we performed immunostaining using a previously characterized anti-ASIC1 (DKG) antibody [69] and an anti-AU1-ASIC3 antibody. Cells transfected with either ASIC1a or ASIC3 alone served as controls for antibody specificity. As shown in Figure 1A, for the co-transfection group, most of the ASIC1a-expressing cells were also positive for AU1-ASIC3 staining. Furthermore, to determine if ASIC1a and ASIC3 formed a heteromeric complex, we performed similar transfections of ASIC1a and ASIC3 to CHO cells, lysed the cells, and conducted immunoprecipitation using an ASIC1 or AU1-ASIC3 antibody, and analyzed total lysate and immunoprecipitated fractions by Western blot. ASIC1a and ASIC3 co-precipitated each other (Figure 1B). These results demonstrate that ASIC1a and ASIC3 are within the same complex in the CHO cells.

#### Co-Overexpression of 1:2, But not 2:1 Ratio of ASIC1a and ASIC3 cDNA Revealed a Profound Response to Zinc

**Figure 2:**
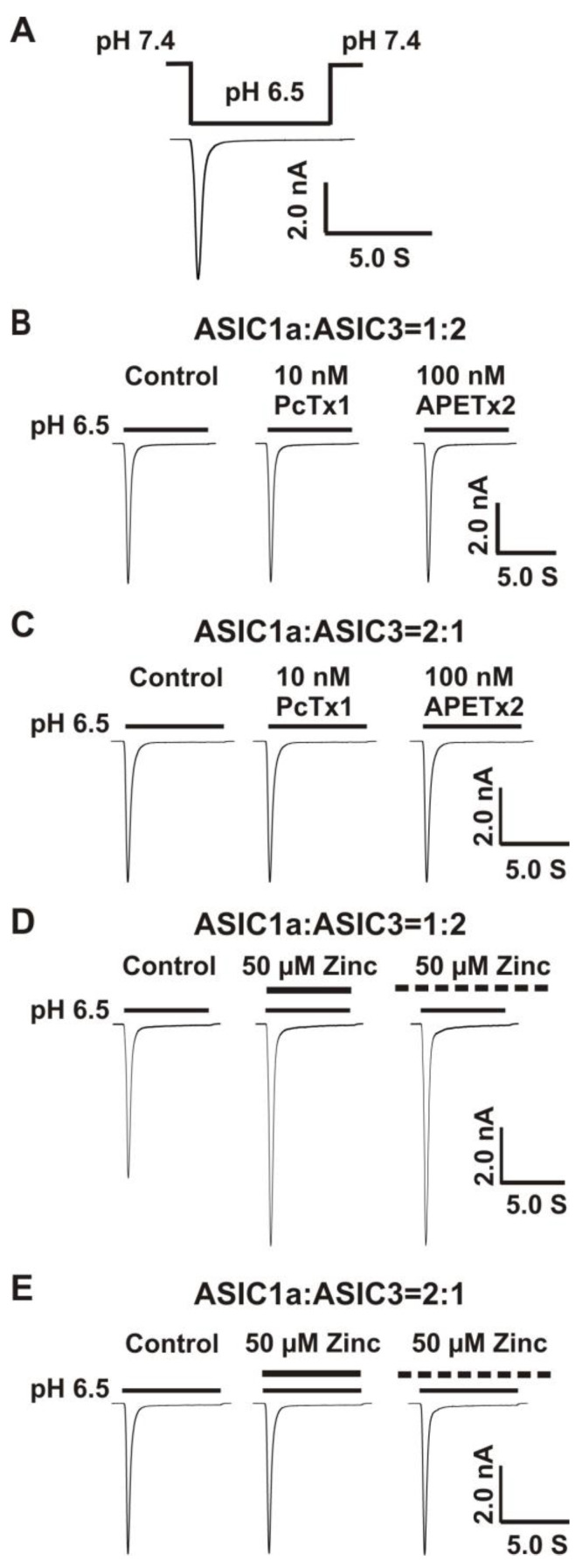
Co-overexpression of 1:2, but not 2:1 ratio of ASIC1a and ASIC3 cDNA revealed a profound response to zinc. (**A**) Activation of heteromeric ASIC1a/3 channels by fast perfusion for a drop in pH from 7.4 to 6.5 on CHO cell expressing both ASIC1a and ASIC3 subunits. The perfusion time for low pH value (e.g., 6.5) is 7 s; (**B**) Representative traces show that PcTx1 (10 nM) and APETx2 (100 nM) have no effects on the heteromeric ASIC1a/3 currents using a 1:2 ratio of ASIC1a and ASIC3, n = 5; (**C**) Representative traces show that PcTx1 (10 nM) and APETx2 (100 nM) also have no effects on the heteromeric ASIC1a/3 currents using a 2:1 ratio of ASIC1a and ASIC3, n = 5; (**D**) Co-application and pretreatment with zinc at 50 µM significantly potentiated the currents of heteromeric ASIC1a/3 using a 1:2 ratio of ASIC1a and ASIC3 (the same cell as Figure 2B), n = 5; (**E**) Co-application and pretreatment with zinc at 50 µM had no effects on the currents of heteromeric ASIC1a/3 using a 2:1 ratio of ASIC1a and ASIC3 (the same cell as Figure 2C), n = 5. Dashed black line represents pretreatment with zinc in pH 7.4 extracellular solution (2 min duration).

Previous studies have shown that ASIC1a and ASIC3 can form functional heteromeric ASIC1a/3 channels [64,65,66,67,68]; however, the ratio of ASIC1a and ASIC3 to form functional channels remains uncertain. ASIC channels are trimers [5]. Two possibilities exist for ASIC1a and ASIC3 to form functional channels. One possibility is the 1:2 ratio of ASIC1a and ASIC3 and another possibility is the 2:1 ratio of ASIC1a and ASIC3. In order to determine if both ratios could form functional heteromeric ASIC1a/3 channels, whole-cell patch-clamp recording was applied to record the currents of heteromeric ASIC1a/3 channels triggered by pH drops. The protocol for activation of heteromeric ASIC1a/3 by a drop in pH from 7.4 to 6.5 is shown in Figure 2A. We recorded the currents in CHO cells overexpressing both ASIC1a and ASIC3 cDNA with the two ratios, as shown in Figure 2B–E. To exclude the possibilities that only homomeric ASIC1a or only homomeric ASIC3 were expressed, selective homomeric ASIC1a inhibitor PcTx1 at a concentration of 10 nM and selective homomeric ASIC3 inhibitor APETx2 at a concentration of 100 nM were used, respectively. At the given concentration, both of the inhibitors did not affect the currents of heteromeric ASIC1a/3 channels triggered by drops in pH from 7.4 to 6.5 under either the 1:2 ratio or the 2:1 ratio of ASIC1a and ASIC3 (Figure 2B,C). To examine if zinc regulated the heteromeric ASIC1a/3 channels, zinc at a concentration of 50 µM was applied to heteromeric ASIC1a/3 channels. As shown in Figure 2D,E, zinc potentiated the currents of heteromeric ASIC1a/3 channels with a 1:2 ratio of ASIC1a and ASIC3, but not a 2:1 ratio of ASIC1a and ASIC3 under both channel open and closed states. Therefore, we conducted the patch-clamp recordings of heteromeric ASIC1a/3 currents with a 1:2 ratio of ASIC1a and ASIC3 in the rest of the studies. Our results suggest that ASIC1a and ASIC3 can form functional channels, and zinc potentiates the currents of heteromeric ASIC1a/3 (1:2 ratio, but not 2:1 ratio) channels under both open and closed states of the channel.

### 3.2. Co-Application of Zinc Potentiated Heteromeric ASIC1a/3 Currents with pH Dependence

#### 3.2.1. Co-Application of Zinc Potentiated the Peak and Sustained Component of ASIC1a/3 Currents with pH Dependence

**Figure 3:**
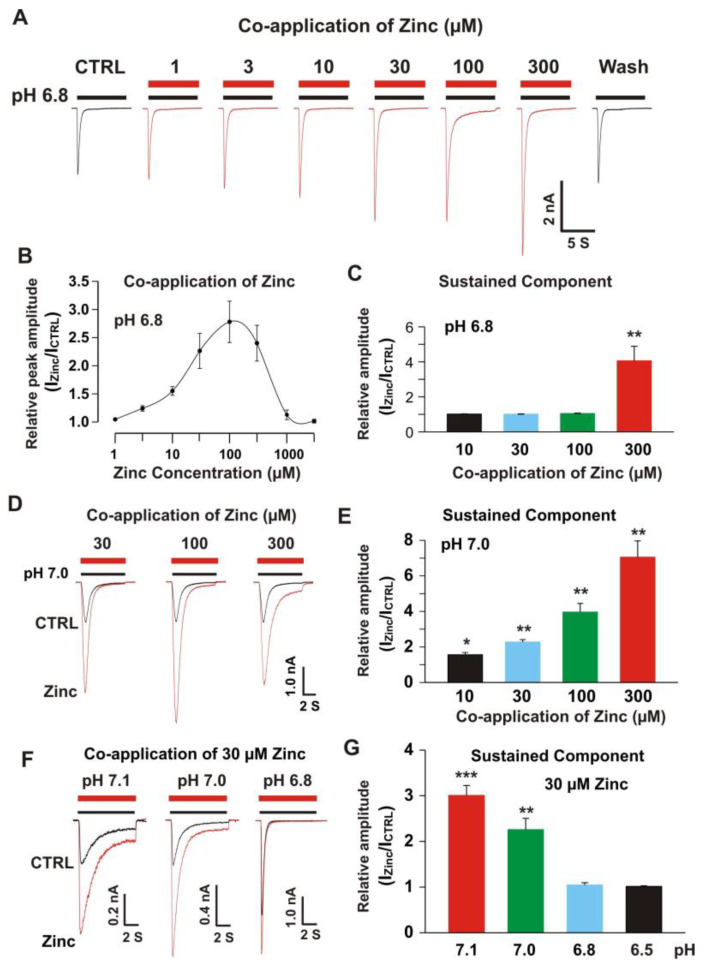
Co-application of zinc potentiated the peak and sustained component of heteromeric ASIC1a/3 currents with pH dependence. (**A**) Representative traces show that zinc dose-dependently enhanced the currents of heteromeric ASIC1a/3 channels recorded on a CHO cell expressing both ASIC1a and ASIC3 subunits; (**B**) Dose-dependent curve of co-application of zinc on heteromeric ASIC1a/3 channels. The curve revealed a bell shape. The EC_50_ for zinc concentration between 1 and 100 µM was 26.0 ± 3.2 µM (*n* = 10) and the EC_50_ for zinc concentration between 100 and 1000 µM was 343.2 ± 49.0 µM (*n* = 10); (**C**) Statistical data show that co-application of zinc at a concentration of 300 µM, but not 10, 30, and 100 µM increase the sustained component of heteromeric ASIC1a/3 currents by a drop in pH from 7.4 to 6.8 (*n* = 12); (**D**) Representative traces show that co-application of zinc at concentrations of 30, 100, and 300 µM potentiate both peak amplitude and sustain component of heteromeric ASIC1a/3 currents by a drop in pH from 7.4 to 7.0; (**E**) Statistical data show that co-application of zinc dose-dependently increase sustained component of heteromeric ASIC1a/3 currents by a drop in pH from 7.4 to 7.0 (*n* = 8); (**F**) Representative traces show that co-application of zinc at a concentration of 30 µM potentiate the heteromeric ASIC1a/3 currents triggered by pH drops from 7.4 to 7.1, 7.0, and 6.8, respectively; (**G**) Statistical data show that co-application of zinc at a concentration of 30 µM enhance the sustained component of heteromeric ASIC1a/3 currents by pH drops from 7.4 to 7.1 and 7.0, but not 6.8 and 6.5 (*n* = 6 to 10). Whole-cell patch-clamp recording was performed. Data are presented as mean ± SEM. CTRL, control; I_CTRL_, ASIC current without any treatment; I_Zinc_, ASIC current by zinc treatment. * *p* < 0.05 and ** *p* < 0.01.

Our previous studies have shown that co-application of zinc at concentrations between 1 and 300 µM did not have a significant effect on homomeric ASIC3 currents [22], but co-application of 300 µM zinc inhibited the homomeric ASIC1a currents [68]. Zinc also potentiated the ASIC1a/3 currents in an open state of the channel (Figure 2D). Next, we examined the dose response of zinc on heteromeric ASIC1a/3 currents triggered by a drop in pH from 7.4 to 6.8 under voltage patch-clamp recording from CHO cells expressing both ASIC1a and ASIC3. The zinc application at concentrations between 1 to 300 µM to the ASIC1a/3 channels was with a pH drop (e.g., 7 s duration during a drop in pH from 7.4 to 6.8). Different from the effects of co-application of zinc on homomeric ASIC1a or homomeric ASIC3 currents, co-application of zinc revealed a profound potentiation of peak amplitude of heteromeric ASIC1a/3 currents from 3.0 to 300 µM dose dependently (Figure 3A). The dose-response curve was constructed with a bell shape with two EC_50s_. One EC_50_ during ranges of 1 to 100 µM zinc is 26.0 ± 3.2 µM and another EC_50_ during ranges of zinc co-application between 100 and 1000 µM is 343.2 ± 49.0 µM (Figure 3B). Co-application of zinc at a concentration of 300 µM, but not 10, 30 or 100 µM, also enhanced the sustained component of the heteromeric ASIC1a/3 by activation of the heteromeric channel from a drop in pH from 7.4 to 6.8 (Figure 3C). However, co-application of zinc at concentrations from 10, 30, 100, and 300 µM, enhanced the sustained component of the heteromeric ASIC1a/3 by a drop in pH from 7.4 to 7.0 (Figure 3D,E). Thus, we further explored the pH dependence of co-application of zinc at a concentration of 30 µM (Figure 3F,G). Co-application of zinc at a concentration of 30 µM potentiated the sustained component of heteromeric ASIC1a/3 currents triggered by pH drops from 7.4 to 7.1 and 7.0, but not 6.8 and 6.5 (Figure 3G). Collectively, our data suggest that co-application of zinc increases both the peak and the sustained component of heteromeric ASIC1a/3 currents in an open state of the channel and this enhancement is pH dependent.

#### 3.2.2. Co-Application of Zinc Shifted pH-Dependent Curve of Heteromeric ASIC1a/3 Channels

**Figure 4:**
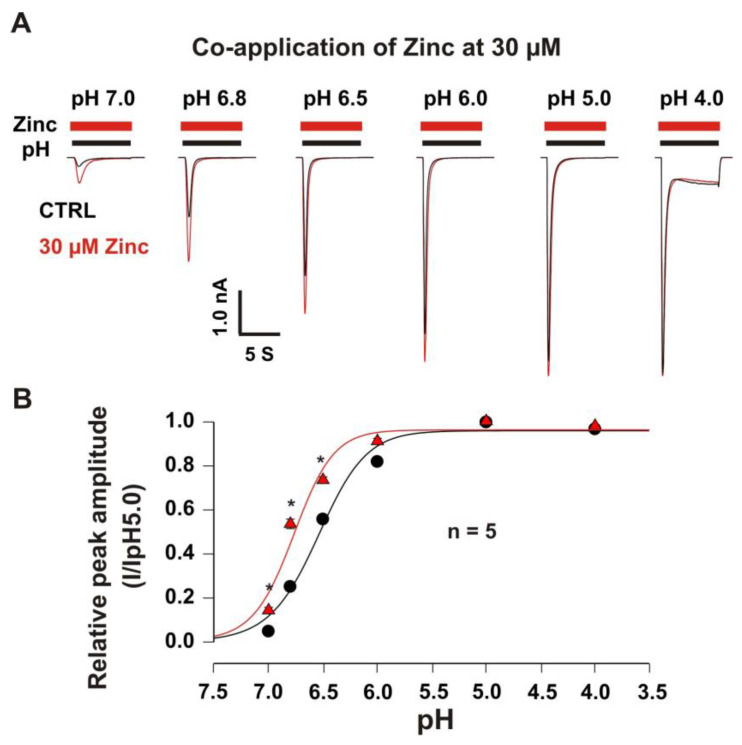
Co-application of zinc at a concentration of 30 µM leftward shifted pH dose–response curve of heteromeric ASIC1a/3 currents. (**A**) Representative traces show that co-application of zinc at a concentration of 30 µM potentiated heteromeric ASIC1a/3 currents with pH dependence; (**B**) Co-application of zinc at a concentration of 30 µM shows a leftward shift of the pH_50_ from 6.54 ± 0.05 without co-application of zinc to 6.77 ± 0.05 with co-application of zinc at a concentration of 30 µM (*p* < 0.05, *n* = 5). Whole-cell patch-clamp recording was performed. Relative peak amplitude with or without zinc treatment was measured by ASIC current (I) on a drop in pH from 7.4 to 7.0, 6.8, 6.5, 6.0, 5.0, or 4.0 divided by ASIC current (I_pH5.0_) on a drop in pH from 7.4 to 5.0. Data are presented as mean ± SEM. CTRL, control. * *p* < 0.05.

In order to explore the pH dependence of activation, the pH dose-response curves with and without zinc were constructed. As shown in Figure 4A,B, the potentiation of the heteromeric ASIC1a/3 currents by zinc at a concentration of 30 µM revealed pH dependence. Co-application of zinc at a concentration of 30 µM also demonstrated a leftward shift of the pH_50_ from 6.54 ± 0.05 without co-application of zinc to 6.77 ± 0.05 with co-application of zinc at a concentration of 30 µM. The potentiation of the ASIC1a/3 currents by co-application of 30 µM zinc was significant by drops in pH from 7.4 to 7.0, 6.8, and 6.5, but not 6.0, 5.0, and 4.0. Our data suggest that the co-application of zinc leftward shifts the pH-dependent activation of the heteromeric ASIC1a/3 channels, indicating an accelerating opening of the heteromeric ASIC1a/3 channels during a closed state of the channel by zinc treatment.

### 3.3. Pretreatment with Zinc Induced Dual Effects on Heteromeric ASIC1a/3 Currents

**Figure 5:**
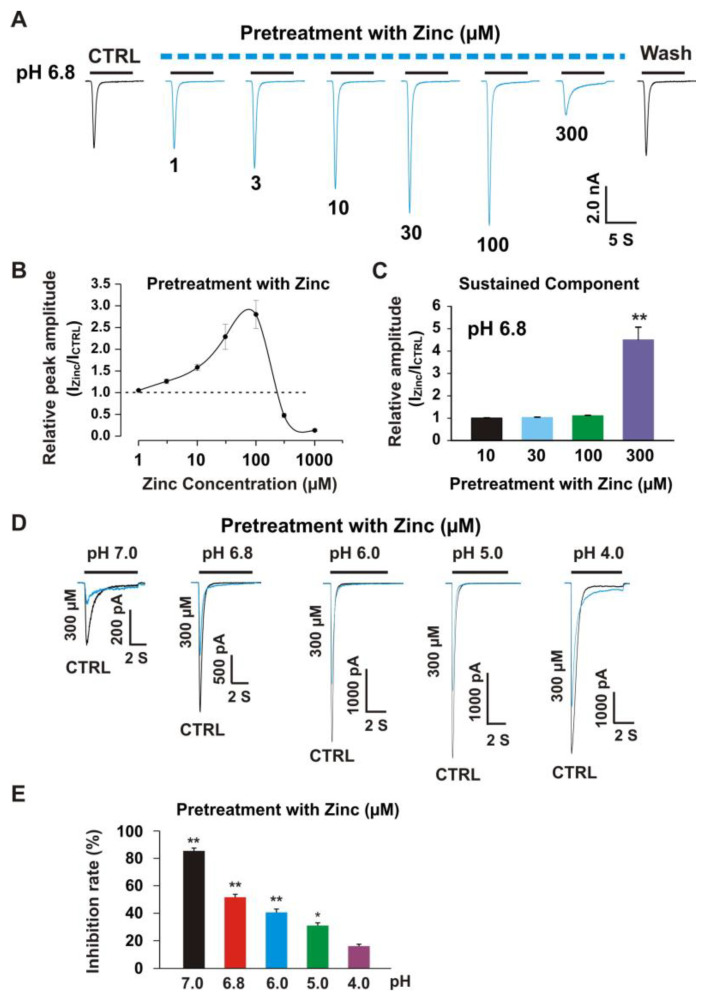
Pretreatment with zinc revealed dual effects on heteromeric ASIC1a/3 currents with pH dependence. (**A**) Representative traces show that pretreatment with zinc (from 3 to 100 µm) was dose-dependently enhanced, whereas pretreatment with zinc at a concentration of 300 µM inhibited the currents of heteromeric ASIC1a/3 channels recorded on a CHO cell expressing both ASIC1a and ASIC3. The dashed blue line represents pretreatment with zinc in pH 7.4 extracellular solution and the solid black line represents co-application of zinc with pH 6.8 (each recoding with 7 s of zinc application); (**B**) Dose-dependent curve of pretreatment with zinc on heteromeric ASIC1a/3 currents. The curve displays a bell-like shape. The EC_50_ for zinc concentrations between 1 and 100 µM was 23.7.0 ± 3.2 µM (*n* = 8) and the EC_50_ for zinc pretreatment between 100 and 250 µM was 127.6 ± 15.6 µM (*n* = 8). The IC_50_ for pretreatment with zinc was 306.0 ± 43.7 µM (*n* = 8); (**C**) Statistical bar graphs show that pretreatment with zinc at a concentration of 300 µM, but not 10, 30, and 100 µM, enhance the sustained component of the heteromeric ASIC1a/3 currents triggered by a pH drop from 7.4 to 6.8; (**D**) Representative traces show that pretreatment with zinc at a concentration of 300 µM inhibit the heteromeric ASIC1a/3 currents triggered by pH drops from 7.4 to 7.0, 6.8, 6.0, and 5.0, but not 4.0, respectively; (**E**) Statistical data show that pretreatment with zinc at a concentration of 300 µM inhibit the peak amplitude of heteromeric ASIC1a/3 currents with pH dependence (*n* = 6 to 10). Whole-cell patch-clamp recording was performed. Data are presented as mean ± SEM. CTRL, control; I_CTRL_, ASIC current without any treatment; I_Zinc_, ASIC current by zinc treatment. * *p* < 0.05 and ** *p* < 0.01.

Our previous studies demonstrated that pretreatment with zinc induced an inhibitory effect on homomeric ASIC3 currents with an IC_50_ of 63 µM [22]. Pretreatment with zinc at a concentration of 300 µM revealed 90% inhibition of peak amplitude of the ASIC3 current [22]. To further investigate if zinc had any effect on heteromeric ASIC1a/3 currents in a closed state of the channel, we pretreated the CHO cells expressing heteromeric ASIC1a/3 at different concentrations of zinc with a duration of 2 min. As shown in Figure 5A, pretreatment with zinc at concentrations between 3.0 and 100 µM showed significant potentiation of heteromeric ASIC1a/3 currents. However, when zinc concentration reached 300 µM, inhibition was found. In the zinc pretreatment studies, we observed changes in the baseline of the recording as higher concentrations of zinc were used, especially zinc at a concentration of 300 µM, suggesting that a larger percentage of the channels were shifted to the closed state with zinc pretreatment. The dose–response curve with potentiation and inhibition was constructed, as shown in Figure 5B. The first EC_50_ for pretreatment with zinc between 1 and 100 µM was 23.7 ± 3.9 µM, which is comparable to co-application of the zinc (26.0 ± 3.2 µM) as shown in Figure 3B. The second EC_50_ during a range of zinc pretreatment between 100 and 250 µM was 127.6 ± 15.6 µM, which was significantly lower than co-application of zinc (see Figure 3B). The IC_50_ for pretreatment with zinc was 306.0 ± 43.7 µM. We also examined the effects on the sustained component of heteromeric ASIC1a/3 currents triggered by a drop in pH from 7.4 to 6.8 in response to pretreatment with different concentrations of zinc, as shown in Figure 5C, and pretreatment with zinc at a concentration of 300 µM potentiated the sustained component of the heteromeric ASIC1a/3 currents. To further explore the inhibition of peak amplitude of the ASIC1a/3 currents by pretreatment with 300 µM zinc, the pH dose response was examined; as shown in Figure 5D,E, and the profound inhibition of peak amplitude of the ASIC1a/3 currents by pretreatment with 300 µM zinc was found on drops in pH from 7.4 to 7.0, 6.8, 6.0, and 5.0, but not 4.0. Taken together, our data suggest that pretreatment with zinc reveals dual effects, which depend on the concentration of zinc. The potentiation and inhibition of the ASIC1a/3 currents by zinc are pH dependent.

### 3.4. Histidine Modifier Diethylpyrocarbonate Blocked the Zinc Effects on Heteromeric ASIC1a/3 Currents

**Figure 6:**
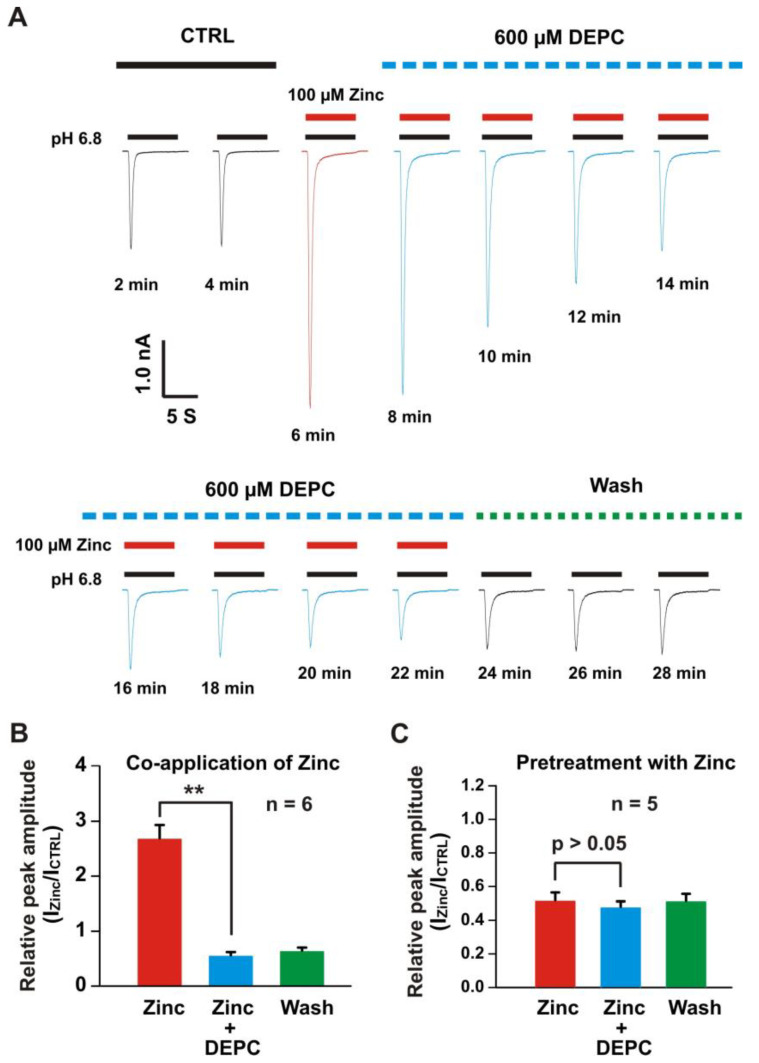
Histidine modifier DEPC blocked the zinc effects on heteromeric ASIC1a/3 channels. (**A**) Representative traces show the time course of the treatment with and without the DEPC (0.6 mM) on potentiation of heteromeric ASIC1a/3 currents by co-application of 100 µM zinc. Treatment with histidine modifying agent DEPC at a concentration of 0.6 mM inhibited the potentiation of heteromeric ASIC1a/3 currents during the incubation. This was recorded in the same cell by different treatments and the current trace was recorded every 2 min. The dashed blue line represents pretreatment with DEPC in the pH 7.4 solution, the dashed green line represents washout with normal pH 7.4 solution, and the solid red and black lines represent 100 µM zinc and pH 6.8 application, respectively (each recoding with 7 s of zinc application); (**B**) Statistical bar graphs show that relative peak amplitude of the ASIC1a/3 currents by zinc potentiation was blocked in the presence of 0.6 mM DEPC (*n* = 6); (**C**) Statistical bar graphs show that relative peak amplitude of the ASIC1a/3 currents by zinc inhibition was blocked in the presence of 0.6 mM DEPC (*n* = 5). Whole-cell patch-clap recording was performed and heteromeric ASIC1a/3 currents were activated by a drop in pH from 7.4 to 6.8. Data are presented as mean ± SEM. CTRL, control; I_CTRL_, ASIC current without any treatment; I_Zinc_, ASIC current by zinc treatment. ** *p* < 0.01.

Our previous studies showed that histidine or cysteine were involved in the zinc modulation of ASICs [22,57,71,72]. Here, we employed the histidine modifier diethylpyrocarbonate (DEPC) to see is it affected the heteromeric ASIC1a/3 currents. As shown in Figure 6A, co-application of zinc at a concentration of 100 µM potentiated the heteromeric ASIC1a/3 currents. Furthermore, we treated the same cell with 0.6 mM DEPC, the time course of treatment of DEPC was displayed, and the DEPC gradually inhibited the ASIC1a/3 currents by co-application of 100 µM zinc. Zinc plus DEPC significantly blocked the potentiation of the channel currents by co-application of zinc only (Figure 6B). We also examined the effect of pretreatment with zinc at a concentration of 300 µM, as shown in Figure 6C, and pretreatment with zinc plus DEPC also blocked the inhibition of ASIC1a/3 currents by pretreatment with zinc only. Our data suggest that histidine residues in the extracellular domain of the channel are involved in the modulation of zinc’s effects on heteromeric ASIC1a/3 channels.

### 3.5. Histidine Residues in the Extracellular Domain of ASIC1a Contributed to Inhibitory Effect by Pretreatment with Zinc on Heteromeric ASIC1a/3 Currents

#### 3.5.1. Zinc Had a Similar Effect on Both ASIC1a Histidine Mutant and ASIC1a Wild-Type Control

**Figure 7:**
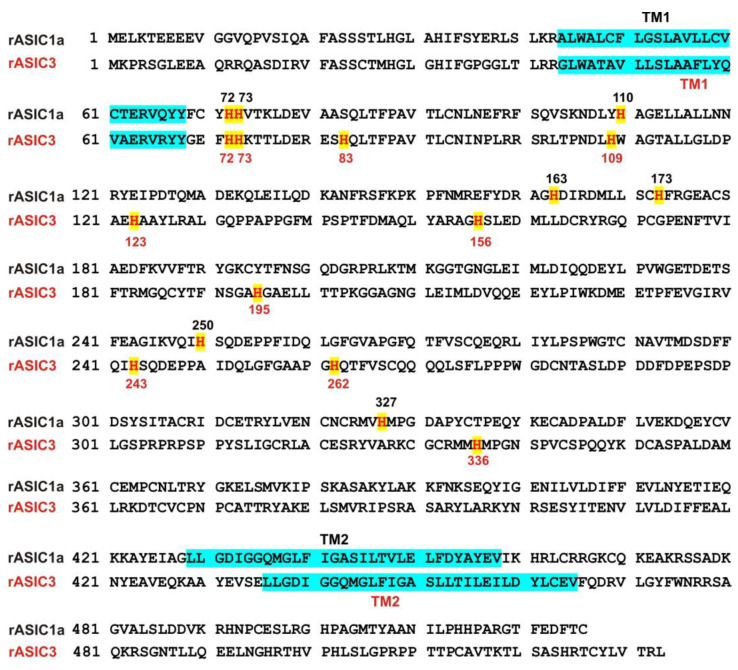
Sequence alignment of rat ASIC1a and rat ASIC3. Sequence alignment of rat ASIC1a (rASIC1a, GenBank ID: 2039366) and rat ASIC3 (rASIC3, GenBank ID: 2352949). Histidine residues in the extracellular domain of rASIC1a and rASIC3 are marked with a yellow color; 7 histidine residues are found in the extracellular domain of rASIC1a and 10 histidine residues are found in the extracellular domain of rASIC3. Transmembrane domain 1 (TM1) and transmembrane domain 2 (TM2) are marked with a dark blue color.

**Figure 8:**
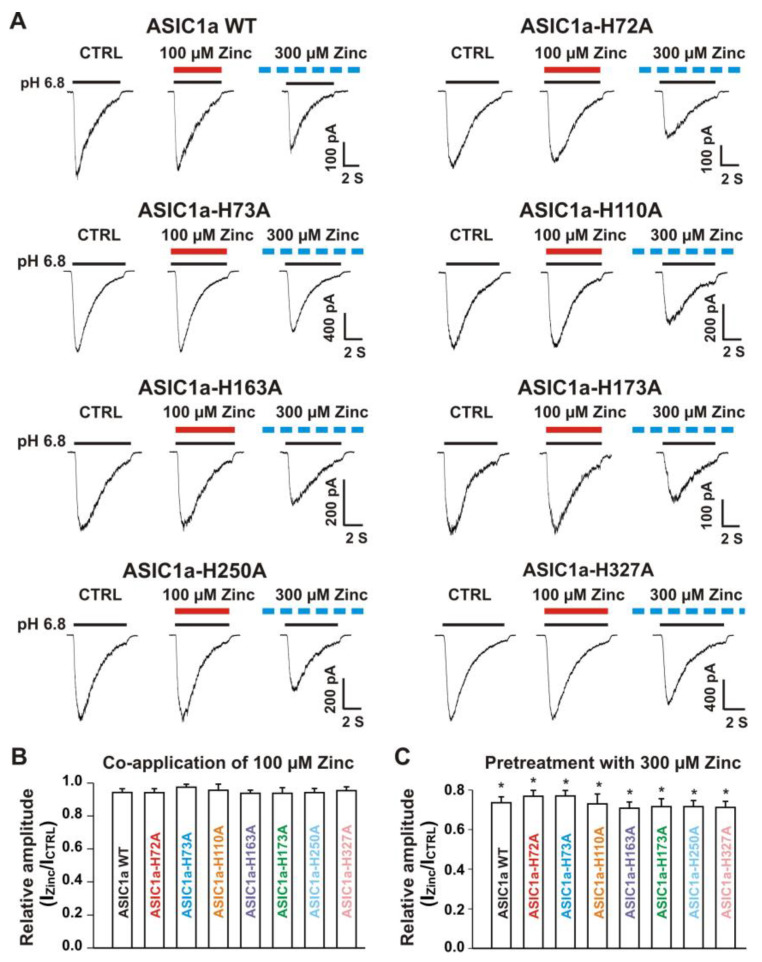
Zinc effects on currents from ASIC1a WT and ASIC1a mutants of histidine residues in the extracellular domain of ASIC1a. (**A**) Representative traces show that co-application of zinc at a concentration of 100 µM had no effect on peak amplitude of the currents from ASIC1a WT, ASIC1a-H72A, ASIC1a-H73A, ASIC1a-H110A, ASIC1a-H163A, ASIC1a-H173A, ASIC1a-H250A, and ASIC1a-H327A; whereas pretreatment with zinc at a concentration of 300 µM displayed an inhibitory effect on peak amplitude of the currents from ASIC1a WT, ASIC1a-H72A, ASIC1a-H73A, ASIC1a-H110A, ASIC1a-H163A, ASIC1a-H173A, ASIC1a-H250A, and ASIC1a-H327A. The solid red and black lines represent 100 µM zinc and pH 6.8 application, respectively (each recoding with 7 s of zinc and pH 6.8 application). The dashed blue line represents pretreatment with 300 µM zinc in the pH 7.4 solution with a duration of 2 min. (**B**,**C**) Statistical bar graphs show relative peak amplitudes of the ASIC1a/3 currents by co-application of zinc (B, *n* = 8 to 10) and pretreatment with zinc (C, *n* = 6 to 10). There was no significant difference for zinc inhibition among ASIC1a WT and their histidine mutants (*p* > 0.05, ANOVA). For ASIC1a mutation, histidine residues in the extracellular domain of ASIC1a were replaced with alanine. Whole-cell patch-clap recording was performed and currents from homomeric ASIC1a WT and its histidine mutants were activated by a drop in pH from 7.4 to 6.8. Data are presented as mean ± SEM. CTRL, control. * *p* < 0.05 (t-test).

Our above results suggest that histidine residues in the extracellular domain of the heteromeric ASIC1a/3 channels contribute to the zinc effects. In order to determine which histidine residue is involved in the modulation of zinc effect, we performed systematic mutagenesis of histidine residues in the extracellular domain of the ASIC1a subunit (Figure 7) and seven histidine residues in the extracellular domain of ASIC1a subunit were found (histidine 72, 73, 110, 163, 173, 250, and 327). All histidine residues were replaced with alanine (i.e., H72A, H73A, H110A, H163A, H173A, H250A, and H327A). All mutants showed a normal response to pH drops with similar pH_50_ as the ASIC1a wild-type (WT) control (see Supplementary Table S1). As shown in Figure 8A,B, co-application of zinc at a concentration of 100 µM did not affect the ASIC currents triggered by a drop in pH from 7.4 to 6.8 in both ASIC1a WT and all mutants. Furthermore, pretreatment with 300 µM zinc slightly inhibited the currents on both ASIC1a WT and all histidine mutants (Figure 8A and C, *p* < 0.05). Inhibition of the currents from all ASIC1a mutants did not reveal differences with the ASIC1a WT control (*p* > 0.05). Together, our data suggest that all histidine mutants of the ASIC1a show similar responses to pH activation, and zinc reveals similar responses on currents recorded from ASIC1a WT and all histidine mutants.

#### 3.5.2. Histidine 72 and 73 in the Extracellular ASIC1a Domain Responsible for Inhibition of Heteromeric ASIC1a/3 Channels by Pretreatment with Zinc

**Figure 9:**
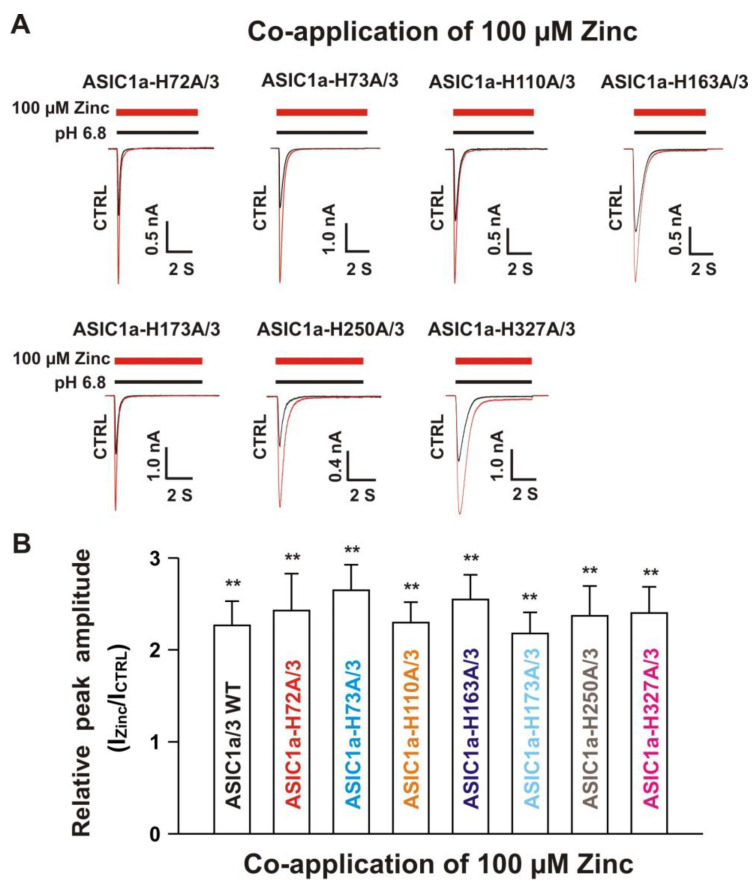
Co-application of zinc potentiated the currents of ASIC1a histidine mutant co-expressed with ASIC3 WT. (**A**) Representative traces show that co-application of zinc at a concentration of 100 µM potentiated the peak amplitude of the currents recorded from heteromeric ASIC1a-H72A/3, ASIC1a-H73A/3, ASIC1a-H110A/3, ASIC1a-H163A/3, ASIC1a-H173A/3, ASIC1a-H250A/3, and ASIC1a-H327A/3; (**B**) Statistical bar graphs show relative peak amplitude of the currents from ASIC1a/3 WTs and its histidine mutant/3 as mentioned above by co-application of 100 µM zinc (*n* = 8 to 15). There was no significant difference for zinc potentiation among ASIC1a/3 WTs with their histidine mutants co-expressed with ASIC3 WT (*p* > 0.05, ANOVA). For ASIC1a mutation, histidine residues in the extracellular domain of ASIC1a were replaced with alanine. Whole-cell patch-clap recording was performed and currents from heteromeric ASIC1a/3 WTs and ASIC1a histidine mutant/3 were triggered by a drop in pH from 7.4 to 6.8. Data are presented as mean ± SEM. CTRL, control; I_CTR_, ASIC current without any treatment; I_Zinc_, ASIC current by zinc treatment. ** *p* < 0.01 (t-test).

**Figure 10:**
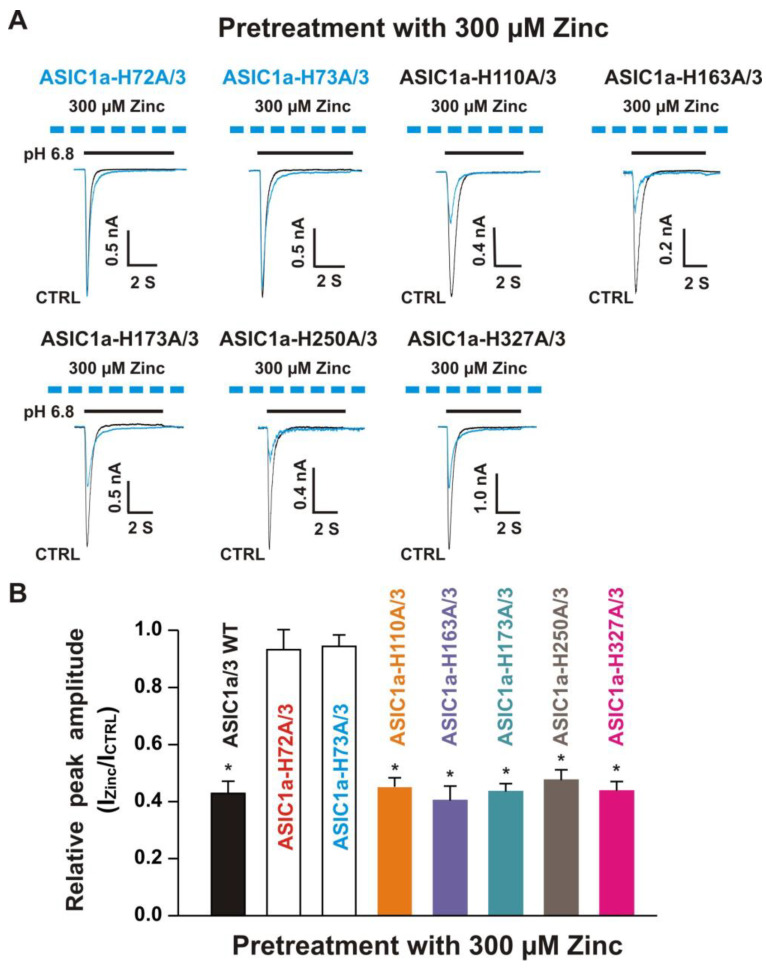
Effects by pretreatment with 300 µM zinc on currents recorded from ASIC1a histidine mutant co-expressed with ASIC3 WT on CHO cells. (**A**) Representative traces show that pretreatment with zinc at a concentration of 300 µM inhibited the currents recorded from heteromeric ASIC1a-H110A/3, ASIC1a-H163A/3, ASIC1a-H173A/3, ASIC1a-H250A/3, and ASIC1a-H327A/3, but not ASIC1a-H72A/3 and ASIC1a-H73A/3. The dashed blue line represents pretreatment with 300 µM zinc in the pH 7.4 solution with a duration of 2 min; (**B**) Statistical bar graphs show relative peak amplitude of the currents from ASIC1a/3 WTs and its histidine mutant/3 as mentioned above by pretreatment with 300 µM zinc (*n* = 7 to 15). Pretreatment with or without 300 µM zinc did not affect the peak amplitude of the currents recorded from ASIC1a-H72A/3 and ASIC1a-H73A/3 (*p* > 0.05, t-test). There was no significant difference for zinc inhibition among ASIC1a/3 WTs, ASIC1a-H110A/3, ASIC1a-H163A/3, ASIC1a-H173A/3, ASIC1a-H250A/3, and ASIC1a-H327A/3 (*p* > 0.05, ANOVA). Whole-cell patch-clap recording was performed and currents from heteromeric ASIC1a/3 WTs and ASIC1a histidine mutant co-expressed with ASIC3 on CHO cells were triggered by a drop in pH from 7.4 to 6.8. Data are presented as mean ± SEM. CTRL, control; I_CTRL_, ASIC current without any treatment; I_Zinc_, ASIC current by zinc treatment. * *p* < 0.05 (*t*-test).

Next, we co-overexpressed each ASIC1a histidine mutant with homomeric ASIC3 WT in CHO cells and examined their effects on currents from ASIC1a histidine mutants co-expressed with ASIC3 WT in response to zinc. Due to co-expression, four possibilities exist. One possibility is only the expression of an ASIC1a histidine mutant, another possibility is only the expression of ASIC3 WT, and the last two possibilities are co-expressions of both an ASIC1a histidine mutant and an ASIC3 WT with different ratios. The homomeric ASIC1a and three channels have different kinetics, especially activation of the channel by a drop in pH from 7.4 to 6.8 [20,22]. Considering only expression of ASIC1a, it is easily observed by the kinetics and also can be significantly blocked by the selective ASIC1a blocker PcTx1 at a concentration of 10 nM [20]. Our above observation provide criteria to distinguish homomeric ASIC3 from ASIC1a mutant/3 by application of zinc with two concentrations with different treatments, i.e., one approach is potentiation of the current by co-application of 100 µM zinc and the other approach is inhibition of the current by pretreatment with 300 µM zinc in the same cell. As shown in Figure 9A, co-application of zinc at a concentration of 100 µM induced potentiation of the currents triggered by a drop in pH from 7.4 to 6.8 on ASIC1a-H72A/3, ASIC1a-H73A/3, ASIC1a-H110A/3, ASIC1a-163A/3, ASIC1a-H173A/3, ASIC1a-H250A/3, and ASIC1a-H327A/3, which were comparable to the ASIC1a/3 WT controls (Figure 9B). Furthermore, pretreatment with zinc at a concentration of 300 µM induced profound inhibition in all ASIC1a histidine mutants co-expressed with ASIC3 WT, except ASIC1a-H72A/3 and ASIC1a-H73A/3, as shown in Figure 10A,B. Taken together, these results suggest that histidine 72 and 73 in the extracellular domain of ASIC1a are responsible for the inhibition of heteromeric ASIC1a/3 channels by pretreatment with zinc.

### 3.6. Effects of Zinc on Heteromeric ASIC1a/3 Currents by Mutation of Histidine Residues in the Extracellular Domain of ASIC3

#### 3.6.1. Zinc Had a Similar Effect on Both ASIC3 Histidine Mutant and ASIC3 WT Control

**Figure 11:**
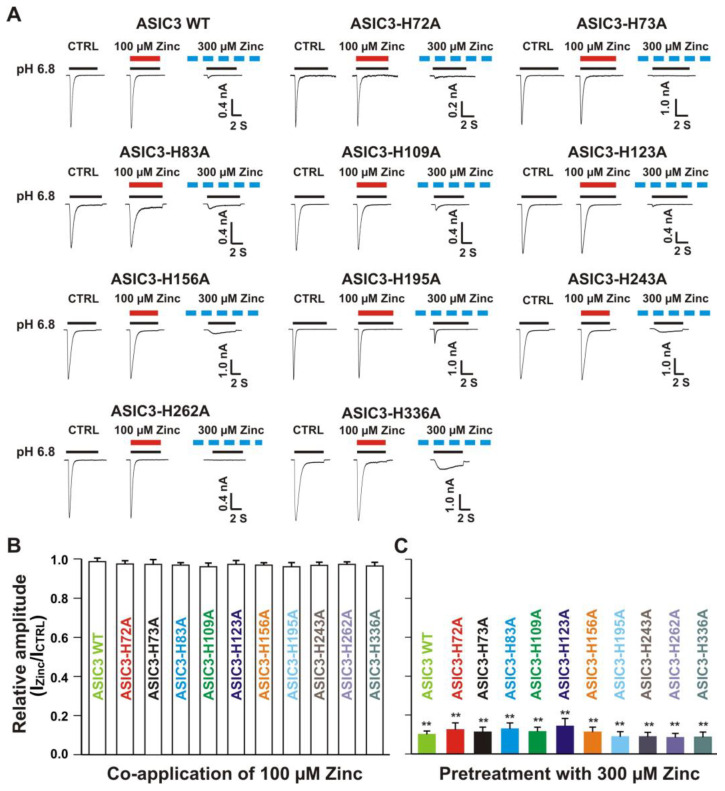
Zinc effects on currents from ASIC3 WT and ASIC3 mutants of histidine residues in the extracellular domain of ASIC3. (**A**) Representative traces show that co-application of zinc at a concentration of 100 µM had no effect on peak amplitude of the currents recorded from ASIC3 WT, ASIC3-H72A, ASIC3-H73A, ASIC3-H83A, ASIC3-H109A, ASIC3-H123A, ASIC3-H156A, ASIC3-H195A, ASIC3-H243A, ASIC3-H262A, and ASIC3-H336A; whereas pretreatment with zinc at a concentration of 300 µM showed profound inhibition on currents from ASIC3 WT, ASIC3-H72A, ASIC3-H73A, ASIC3-H83A, ASIC3-H109A, ASIC3-H123A, ASIC3-H156A, ASIC3-H195A, ASIC3-H243A, ASIC3-H262A, and ASIC3-H336A. The solid red and black lines represent 100 µM zinc and pH 6.8 application, respectively (each recoding with 7 s of zinc and pH 6.8 application). The dashed blue line represents pretreatment with 300 µM zinc in the pH 7.4 solution with a duration of 2 min. (**B** and **C**) Statistical bar graphs show relative peak amplitudes of the currents by co-application of zinc (B, *n* = 7 to 10) and pretreatment with zinc on ASIC3 WT and their histidine mutants expressed on CHO cells as mentioned above (C, *n* = 6 to 10). There was no significant difference on currents by co-application of zinc or pretreatment with zinc (inhibitory effect) among ASIC3 WT and their histidine mutants (*p* > 0.05, ANOVA). For ASIC3 mutation, histidine residues in the extracellular domain of ASIC3 were replaced with alanine. Whole-cell patch-clap recording was performed and currents of ASIC3 and its mutants were activated by a drop in pH from 7.4 to 6.8. Data are presented as mean ± SEM. CTRL represents control. I_CTRL_, ASIC current without any treatment; I_Zinc_, ASIC current by zinc treatment. ** *p* < 0.01 (*t*-test).

We further conducted systematic mutagenesis of histidine residues in the extracellular domain of homomeric ASIC3 subunit. As shown in Figure 7, 10 histidine residues in the extracellular domain of the ASIC3 subunit were found (histidine 72, 73, 83, 109, 123, 156, 195, 243, 262, and 336). Similar to homomeric ASIC1a mutation as mentioned above, histidine residues were replaced with alanine (i.e., H72A, H73A, H83A, H109A, H123A, H156A, H195A, H243A, H262A, and H336A). All mutants showed a normal response to pH drops with similar pH_50_ as the ASIC3 WT (see Supplementary Table S2). As shown in Figure 11A,B, co-application of zinc at a concentration of 100 µM did not change the peak amplitude of the ASIC currents triggered by a drop in pH from 7.4 to 6.8 in both ASIC3 WT and all ASIC3 mutants (*p* > 0.05). Furthermore, pretreatment with 300 µM zinc significantly inhibited the currents on both ASIC3 WT and all ASIC3 histidine mutants (*p* < 0.05), and inhibition of the currents from all ASIC3 mutants did not display a difference with ASIC3 WT control (*p* > 0.05). Together, our data suggest that all mutants display a similar response to pH activation and zinc reveals a similar response on currents from ASIC3 WT and ASIC3 histidine mutants.

#### 3.6.2. Histidine 72, 73, and 83 in the Extracellular Domain of ASIC3 Responsible for Zinc Effects on Heteromeric ASIC1a/3 Channels

**Figure 12:**
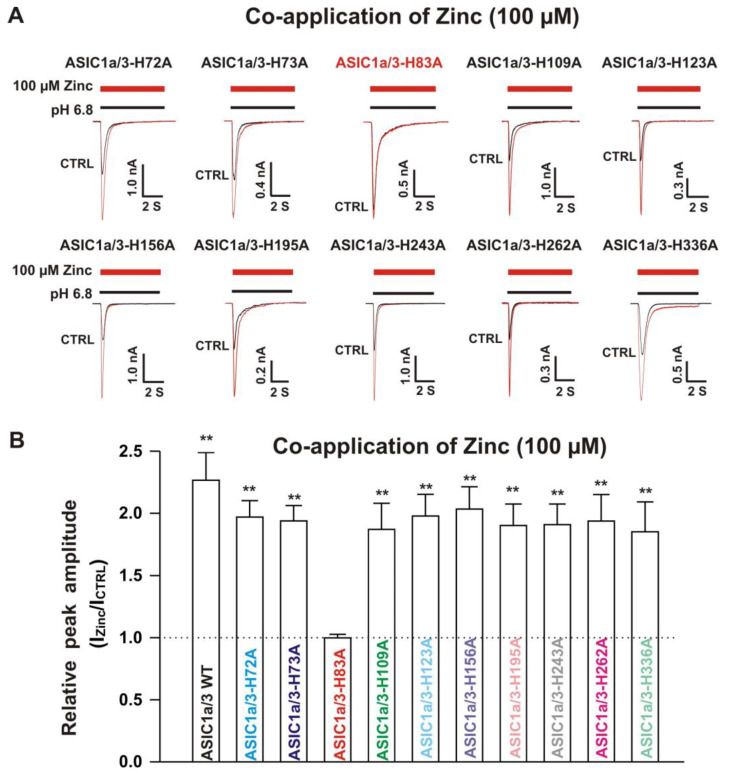
Effects by co-application of zinc on currents from ASIC1a WT co-expressed with ASIC3 histidine mutants on CHO cells. (**A**) Representative traces show that co-application of zinc at a concentration of 100 µM potentiated the peak amplitude of the currents recorded from heteromeric ASIC1a/3-H72A, ASIC1a/3-H73A, ASIC1a/3-H109A, ASIC1a/3-H123A, ASIC1a/3-H156A, ASIC1a/3-H195A, ASIC1a/3-H243A, ASIC1a/3-H262A, and ASIC1a/3-H336A, but not ASIC1a/3-H83A; (**B**) Statistical bar graphs show relative peak amplitude of the currents from ASIC1a/3 WTs and its histidine mutant co-expressed with ASIC3 WT as mentioned above by co-application of 100 µM zinc (*n* = 7 to 15). Co-application of 100 µM zinc did not affect the current without zinc application on ASIC1a/3-H83A (*p* > 0.05, t-test). There was no significant difference for zinc potentiation among ASIC1a/3 WTs with ASIC1a WT co-expressed with ASIC3 histidine mutants except ASIC1a/3-H83A (*p* > 0.05, ANOVA). For ASIC3 mutation, histidine residues in the extracellular domain of ASIC3 were replaced with alanine. Whole-cell patch-clap recording was performed, and currents recorded from heteromeric ASIC1a/3 WTs and ASIC1a WT with ASIC3 histidine mutants were triggered by a drop in pH from 7.4 to 6.8. Data are presented as mean ± SEM. CTRL, control; I_CTRL_, ASIC current without any treatment; I_Zinc_, ASIC current by zinc treatment. ** *p* < 0.01 (t-test).

**Figure 13:**
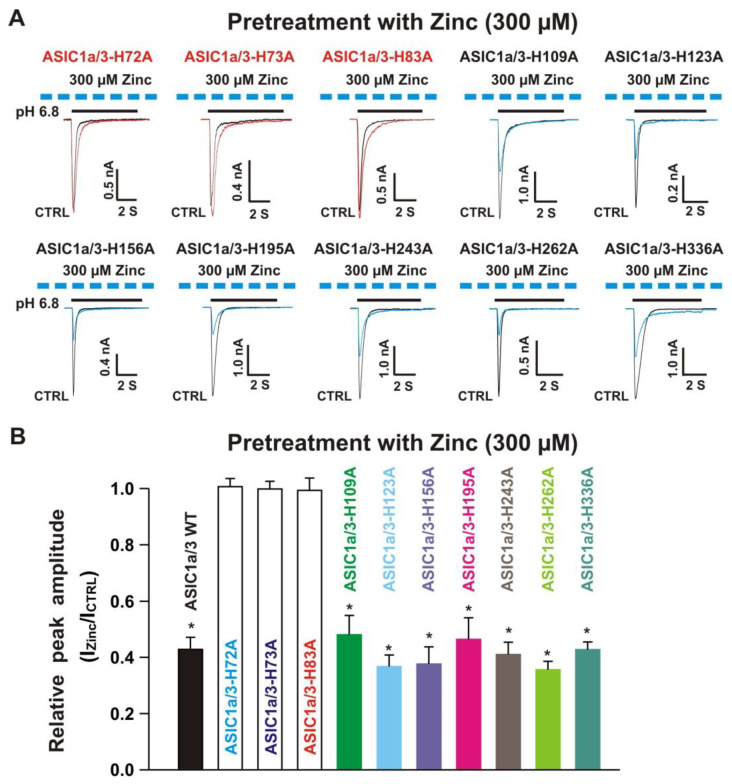
Effects by pretreatment with 300 µM zinc on currents recorded from ASIC1a WT co-expressed with ASIC3 histidine mutants on CHO cells. (**A**) Representative traces show that pretreatment with zinc at a concentration of 300 µM inhibited the peak amplitude of the currents recorded from heteromeric ASIC1a/3-H109A, ASIC1a/3-H123A, ASIC1a/3-H156A, ASIC1a/3-H195A, ASIC1a/3-H243A, ASIC1a/3-H262A, and ASIC1a/3-H336A, but not ASIC1a/3-H72A, ASIC1a/3-H73A, and ASIC1a/3-H83A. The dashed blue line represents pretreatment with 300 µM zinc in the pH 7.4 solution with a duration of 2 min; (**B**) Statistical bar graphs show relative peak amplitudes of the currents by pretreatment with 300 µM zinc from ASIC1a/3 WTs and ASIC1a with ASIC3 histidine mutants as mentioned above (*n* = 7 to 15). Pretreatment with zinc at a concentration of 300 µM did not affect the peak amplitude of the currents without zinc treatment on ASIC1a/3-H72A, ASIC1a/3-H73A, and ASIC1a/3-H83A (*p* > 0.05, t-test). There was no significant difference for zinc inhibition among ASIC1a/3 WT, ASIC1a/3-H109A, ASIC1a/3-H123A, ASIC1a/3-H156A, ASIC1a/3-H195A, ASIC1a/3-H243A, ASIC1a/3-H262A, and ASIC1a/3-H336A (*p* > 0.05, ANOVA). For ASIC3 mutation, histidine residues in the extracellular domain of ASIC3 were replaced with alanine. Whole-cell patch-clap recording was performed, and currents recorded from heteromeric ASIC1a/3 WTs and ASIC1a WT with ASIC3 histidine mutants were triggered by a drop in pH from 7.4 to 6.8. Data are presented as mean ± SEM. CTRL, control; I_CTRL_, ASIC current without any treatment; I_Zinc_, ASIC current by zinc treatment. * *p* < 0.05 (t-test).

Next, we co-expressed each ASIC3 mutant with homomeric ASIC1a WT in CHO cells and determined their effects on ASIC1a/3 mutant currents by zinc. Similar to co-expression of an ASIC1a mutant with ASIC3 WT, four possibilities exist. One possibility is only the expression of homomeric ASIC1a WT, another possibility is only the expression of ASIC3 mutant, and the last two possibilities are co-expressions of both ASIC1a WT and ASIC3 mutant with different ratios. We used similar methods to distinguish one from the other, as mentioned above. As shown in the Figure 12A,B, co-application of zinc at a concentration of 100 µM triggered potentiation of currents by a drop in pH from 7.4 to 6.8 recorded on ASIC1a/3-H72A, ASIC1a/3-H73A, ASIC1a/3-H109A, ASIC1a/3-H123A, ASIC1a/3-H156A, ASIC1a/3-H195A, ASIC1a/3-H243A, ASIC1a/3-H262A, and ASIC1a/3-H336A. However, zinc did not reveal potentiation on currents activated by a drop in pH from 7.4 to 6.8 on ASIC1a/3-H83A. Furthermore, pretreatment with zinc at a concentration of 300 µM significantly inhibited the currents by a drop in pH from 7.4 to 6.8 on ASIC1a/3-H109A, ASIC1a/3-H123A, ASIC1a/3-H156A, ASIC1a/3-H195A, ASIC1a/3-H243A, ASIC1a/3-H262A, and ASIC1a/3-H336A, but not on ASIC1a/3-H72A, ASIC1a/3-H73A, and ASIC1a/3-H83A, as shown in Figure 13A,B. In order to exclude other possibilities of co-expression of ASIC1a/3-H83A, PcTx1 (10 nM) and APETx2 (100 nM) were used. Two inhibitors did not show inhibition of the currents recorded from co-expression of ASIC1a and ASIC3-H83A in CHO cells (data not shown), indicating that co-expression of ASIC1a and ASIC3-H83A formed heteromeric ASIC1a/3-H83A channels. Collectively, these results suggest that ASIC1a/3-H83A contributes to both potentiation and inhibition by zinc, whereas ASIC1a/3-H72A, and ASIC1a/3-H73A are responsible for zinc-mediated inhibition.

## 4. Discussion

ASICs are activated by a drop in pH and different ASIC isoforms exhibit different pH sensitivities [15]. Previous studies have shown that the pH_50_ of the ASIC1a (pH 6.2) and ASIC3 (pH 6.8) homotrimers were different [22,57], ASIC3 homotrimers are more sensitive to protons with respect to ASIC1a homotrimers, as well as to the ASIC1a/3 heterotrimers (pH 6.54 in the present studies, see also Figure 4). In line with this observation, we expect that homomeric ASIC3 is the most activated isoform when stimulating cells at pH 6.8. Studies from the Lazdunski group have shown that zinc revealed an excitatory effect on homomeric ASIC2a and heteromeric ASIC1a/2a channels [58]. Our previous studies have demonstrated that zinc induced an inhibitory effect on homomeric ASIC1a [57], ASIC1b [71,72], and ASIC3 channels [22]. However, whether or not zinc has any effect on heteromeric ASIC1a/3 channels during open and closed states of the channels remains uncertain. In the present studies, we first examined the zinc effects on heteromeric ASIC1a/3 channels. To our surprise, co-application of zinc or pretreatment with zinc at concentrations between 3 and 100 µM induced a dose-dependent potentiation of heteromeric ASIC1a/3 currents triggered by a drop in pH from 7.4 to 6.8 with an EC_50_ of 26 µM, which was different from findings of no effect by co-application of zinc at the same concentration on homomeric ASIC1a or homomeric ASIC3 (see Figure 8 and Figure 11). Furthermore, pretreatment with zinc at a concentration of 300 µM revealed a significant inhibition of heteromeric ASIC1a/3 currents (around 60% inhibition of peak amplitude, see Figure 5), which also showed different degrees of inhibition by zinc (300 µM) on homomeric ASIC1a (about 30% inhibition of peak amplitude, see Figure 8) or homomeric ASIC3 (90% inhibition of peak amplitude, see Figure 11). Furthermore, zinc treatment not only affected the peak amplitude, but also the sustained component of heteromeric ASIC1a/3 currents and this effect was pH dependent.

On the basis of our electrophysiological recordings, we speculate that certain effects of co-expressed currents were due to the direct effect on ASIC1a/3 heterotrimers. Our co-IP result provided a direct support for the existence of ASIC1a/3 heterotrimers. To explore the mechanisms of zinc’s effects on heteromeric ASIC1a/3 channels, the dose response of pH activation was explored. Co-application of zinc induced a leftward shift of the pH activation curve on pH_50_ from 6.54 without zinc treatment to 6.77 with zinc treatment. These results suggest that zinc treatment accelerates the ASIC1a/3 channels open state. Then, we employed the histidine modifier DEPC to further examine the effects of zinc on the channel and we found that DEPC blocked the zinc effects on heteromeric ASIC1a/3 channels. Therefore, we systematically mutated the histidine residues in the extracellular domain of either homomeric ASIC1a or homomeric ASIC3 subunit. We found seven histidine residues in the extracellular domain of the ASIC1a subunit and 10 histidine residues in the extracellular domain of the ASIC3 subunit. All 17 histidine residues were replaced with alanine by point mutation. We identified that histidine residues 72 and 73, in both the ASIC1a and ASIC3 subunits, and histidine 83, in the ASIC3 subunit, are responsible for zinc effects on heteromeric ASIC1a/3 channels. Although the heteromeric ASIC1a/3 channels can be modulated by endogenous and exogenous molecules [23,66,67], there is still no selective pharmacological agent able to identify the heteromeric ASIC1a/3 channels. This study provides alternative evidence to further identify the heteromeric ASIC1a/3 using endogenous biomolecule zinc. Most importantly, we identified histidine residues in the extracellular domain of the ASIC1a/3 channels responsible for zinc-mediated effects. 

To gain structural insight into the requirements of histidine (His) residues identified in the present studies on zinc regulation or modulation of the heterotrimeric ASIC1a/3 channels, we mapped His72 and His73 in rat ASIC1a and rat ASIC3, as well as His83 in rat ASIC3, onto the crystal structure of chicken ASIC1a [5]. Because we used a 1:2 ratio of ASIC1a and ASIC3 in most of our studies, the images shown in Supplementary Figures S1 and S2 assume one subunit in the crystal structure is ASIC1a (shown in grey) and two subunits in the crystal structure are ASIC3 (shown in cyan and yellow). On the basis of the sequence alignment of rat ASIC1a, rat ASIC3, and chicken ASIC1a (Supplementary Figure S3), we made the following mutations in the crystal structure to represent the His residues identified in our present studies (Supplementary Figures S1 and S2). Proline 73 (Pro73) in the crystal structure is mutated to His in all three subunits to represent His72 in rat ASIC1a and rat ASIC3. Threonine 84 (Thr84) in the crystal structure is mutated to His in two of the subunits to represent His83 in rat ASIC3 and to serine (Ser) in one subunit to represent Ser83 in rat ASIC1a. As shown in Supplementary Figure S1, His72 and His73 are situated in the short loop that connects the transmembrane helix 1 and the β1 strand. This short loop is also referred to as the wrist of the ASIC channel [4,5], which connects the extracellular proton sensing part of the channel to the transmembrane pore and it undergoes large conformational rearrangement upon channel opening [4]. His83 (rat ASIC3) and Ser84 (rat ASIC1a) are located in the loop that connect the β1 and β2 strands and sit below the acidic pocket that is responsible for proton sensing of the channel (Supplementary Figure S1) [4,5]. The β1 strand is displaced upon channel opening [4], suggesting that the His83 (ASIC3) and S84 (ASIC1a) likely also undergo conformation change in this process. 

Examination of the His72, His73, and His83 on ASIC structure indicates that they likely contribute to the allosteric pathway for zinc regulation or modulation of the heterotrimeric ASIC1a/3 channels. However, it is not clear whether or not these His residues are directly involved in zinc ion binding. To explore the possibility of His72, His73, and His83 coordination to zinc ions, we looked for other residues such as cysteine (Cys), aspartate (Asp), or glutamate (Glu) that are known to be involved in protein binding to zinc ions near His72, His73, and His83 residues [74]. As shown in Supplementary Figure S2, His72 and His73 in one subunit are close to two acidic residues from the neighboring subunit, Asp78 (rat ASIC3) and Glu426 (rat ASIC3). Together, these four residues could potentially serve as the coordination site for a zinc ion. Additional water molecules can also participate in zinc ion coordination. Moreover, these two acidic residues are also found in rat ASIC1a as Asp78 and Glu419, suggesting this tentative zinc binding site can be formed at all three subunit interfaces. 

Near ASIC3 His83, we found three acid residues on the crystal structure, chicken ASIC1a Glu358, Glu363, and Asp290 (Supplementary Figure S2). These residues correspond to Glu357, Glu362, and Thr289 in rat ASIC1a (Supplementary Figure S3). Although only two of the three acidic residues are conserved in ASIC1a, considering this region is solvent exposed, it is possible that water molecules can also participate in zinc binding at this site. Under the assumption that the ASIC1a and ASIC3 heterotrimeric channels contain one ASIC1a and two ASIC3, in addition to the interface shown in Supplementary Figure S2, which is formed between one ASIC1a and one ASIC3, there is also an interface between two ASIC3 subunits that contains His83 in one subunit. We examined the possibility of this interface also forming a potential zinc binding site. According to sequence alignment (Supplementary Figure S3), chicken Asp290 corresponds to rat ASIC3 Asp282, whereas chicken Glu358 is missing in rat ASIC3, and Glu363 corresponds to Pro369 in rat ASIC3. The fact that only one acidic residue is conserved at this interface suggests this region of the ASIC3/ASIC3 interface is unlikely to form a zinc binding site. This prediction is consistent with the observation that zinc ion has no effect on the homomeric ASIC3 channels in an open state of the channels [22,58]. In our future studies, we plan to test these hypothesized zinc binding sites, as shown in Supplementary Figure S2. Of course, it is also likely that His72, His73, and His83 are not directly involved in zinc binding and their requirements in zinc effects of heterotrimeric ASIC1a/3 channels are achieved through allosteric regulation or modulation by the zinc binding sites in other part of the protein. Further investigations are needed to reveal the structural mechanisms of zinc regulation or modulation of the heterotrimeric ASIC1a/3 channels.

Zinc, the second richest biomolecule in the brain, contributes to physiological and pathological conditions [59,60,61]. It is also involved in neurological diseases [60,61,62,63]. Therefore, it is important to know whether it also regulates or modulates the heteromeric ASIC1a/3 channels, which likely exist in the nervous system. With zinc as a highly present endogenous biomolecule, our studies provided molecular mechanisms of zinc regulation or modulation of heteromeric ASIC1a/3 channels, which could be mechanistically translated for future drug development. 

Changes in extracellular pH occur in the retina and directly affect retinal activity and phototransduction. Several studies have shown that ASIC1a and ASIC3 were widely expressed in retina ganglion cells and played functional roles in the physiological and pathological processes [75,76]. For example, studies from ASIC1a knock-out mice suggested that ASIC1a channels played critical roles in normal retinal activity. ASIC1a channels contribute to gain adaptation to ambient light of the cone pathway, facilitate cone hyperpolarization in brightness, and regulate synaptic transmission of the light-induced visual signal [77]. In disease condition, recent studies have shown that ASIC1a channels revealed protection in ischemia reperfusion in mouse eyes and in the optic nerve crush model in rat [78,79]. Studies from ASIC3 knock-out mice reported that ASIC3 channels were involved in maintaining retinal integrity [76]. Inactivation of ASIC3 enhanced visual transduction and revealed less rod photoreceptor death as compared with WT mice [76].

Given high expressions of ASIC1a and ASIC3 and their roles in the retina, therefore, it is possible to form functional ASIC1a/3 channels. Because zinc can be released during neurotransmission in the retina, it could regulate or modulate ASIC1a/3 activities during the physiological process or disease state. More recently, ASIC1a and ASIC3 have been found in glioblastoma cancer stem cell lines, whether or not they form heteromeric ASIC1a/3 channels is unclear [80]. Therefore, future studies are needed to examine whether heteromeric ASIC1a/3 channels are indeed present in the retina or other tissues, their physiological as well as pathological roles, and regulation/modulation by biomolecule zinc or other endogenous biomolecules.

## 5. Conclusions

In the present study, we demonstrated novel findings of biphasic effects of zinc treatment on heteromeric ASIC1a/3 channels and identified histidine residues in the extracellular domain of the channel responsible for zinc-mediated effects. This study provided a molecular basis for zinc regulation or modulation of the heteromeric ASIC1a/3 channels.

## Supplementary Materials

The following are available online at https://www.mdpi.com/2218-273X/10/9/1264/s1, Table S1: pH_50_ and Hill value of ASIC1a WT and ASIC1a histidine mutants in the extracellular domain of ASIC1a subunit, Table S2: pH_50_ and Hill value of ASIC3 WT and ASIC3 histidine mutants in the extracellular domain of ASIC3 subunit.
