# Supplementary material for: Histidine Residues Are Responsible for Bidirectional Effects of Zinc on Acid-Sensing Ion Channel 1a/3 Heteromeric Channels
Source: Biomolecules. 2020 Sep 2;10(9):1264. doi: 10.3390/biom10091264 (PMC7565092; doi:10.3390/biom10091264)
Supplement: Supplementary file 1 [file biomolecules-10-01264-s001.zip › biomolecules-882257-sup.pdf]

Supplementary Figure 1. Residues H72/H73 in rat ASIC1a, rat ASIC3 and H83 in rat ASIC3 are mapped onto the crystal structure of chicken ASIC1a. The three subunits in the heterotrimer are colored to represent one rASIC1a (grey) subunit and two rASIC3 subunits (cyan and yellow) in the ASIC1a/ASIC3 heterotrimeric channel. Based on the sequence alignment shown in Supplementary Figure 3, in the image shown here, P72 in 2QTS is mutated to H; T84 in subunit 1 is mutated to S; T84 in subunits 2 and 3 are mutated to H. The images are generated using PyMOL. r represents rat in the Figure.

Supplementary Figure 2. Potential Zn^{2+} binding sites on heterotrimeric ASIC1a/3 channel mapped onto a crystal structure of the ASIC1a channel. The three subunits in the heterotrimer are colored to represent one rASIC1a (grey) subunit and two rASIC3 subunits (cyan and yellow) in the ASIC1a/3 heterotrimeric channel. Only sites at one inter-subunit interface are shown. r represents rat; c represent chicken in the Figure. The images are generated using PyMOL.

Supplementary Figure 3. Sequence alignment of rat ASIC1a, rat ASIC3 and the sequence of chicken ASIC1a, which is used to determine the crystal structure of ASIC1a channel. The alignment is performed using the serer PROMAL3D.

Supplementary Figure 1

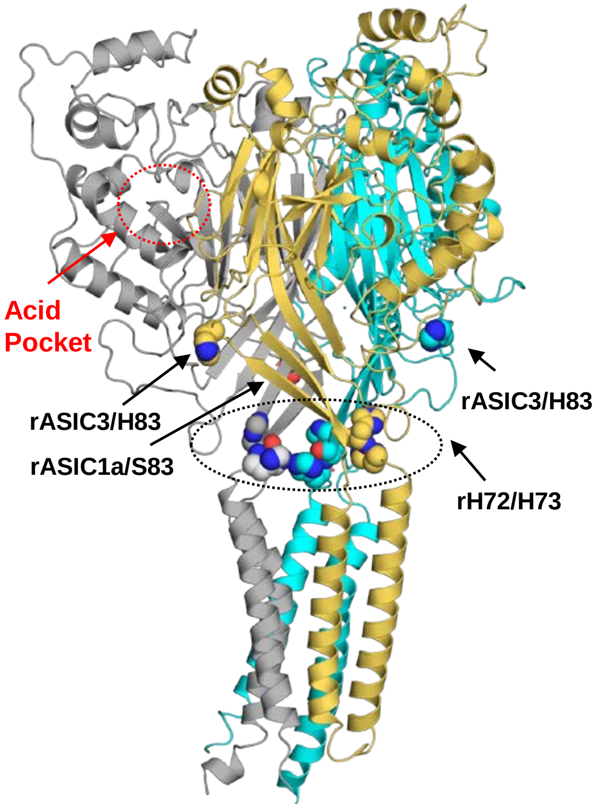

Supplementary Figure 2

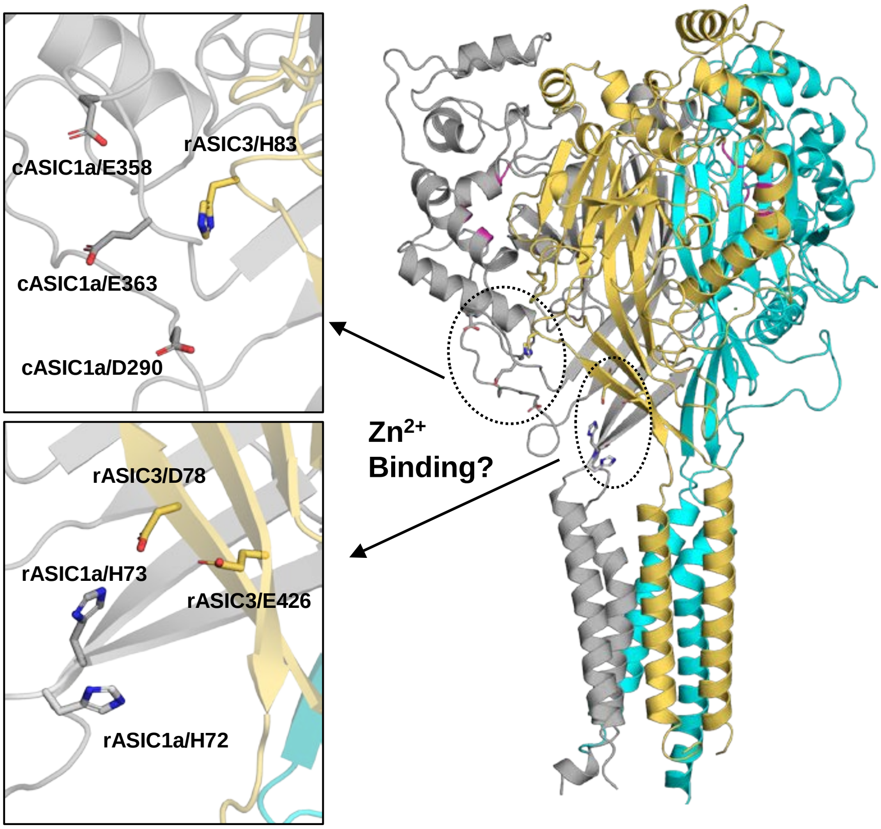

Colored PROMALS3D alignment (sequences in input order)

```
Conservation:        9      99    9   9  9999 9 99  999    9
ratASIC1a        1  -MELKTEEEEVGGVQPVSIQAFASSSTLHGLAHIFSYERL   39
ratASIC3         1  -MKPRSGLEEAQRRQASDIRVFASSCTMHGLGHIFGPGGL   39
chickenASIC1a    1  MMDLKVDEEEVDSGQPVSIQAFASSSTLHGISHIFSYERL   40
Consensus_aa:       .Mc.+s.bEEh...QsssIphFASStThHGltHIFt...L
Consensus_ss:               hhh    hhhhhhhhh      ehhhhe

Conservation:        9 9  99     999        9  99    9 9 999
ratASIC1a       40  SLKRALWALCFLGSLAVLLCVCTERVQYYFCYHHVTKLDE   79
ratASIC3        40  TLRRGLWATAVLLSLAAFLYQVAERVRYYGEFHHKTTLDE   79
chickenASIC1a   41  SLKRVVWALCFMGSLALLALVCTNRIQYYFLYPHVTKLDE   80
Consensus_aa:       oL+RslWAhthh.SLAhhhh.hhpRlpYY..@.H.TpLDE
Consensus_ss:       hhhhhhhhhhhhhhhhhhhhhhhhhhhhhh   eeeeeee

Conservation:            9999999 99 9  9 9    999  99  9  9
ratASIC1a       80  VAASQLTFPAVTLCNLNEFRFSQVSKNDLYHAGELLALLN  119
ratASIC3        80  RESHQLTFPAVTLCNINPLRRSRLTPNDLHWAGTALLGLD  119
chickenASIC1a   81  VAATRLTFPAVTFCNLNEFRFSRVTKNDLYHAGELLALLN  120
Consensus_aa:       ..tppLTFPAVThCNlN.hR.Splo.NDL@@AGphLh.Ls
Consensus_ss:       eee eee   eee     hhhhhhhhhhhhhhhhhhhhhh

Conservation:                      9  9        9     9 9   9
ratASIC1a      120  NRYEIPDTQMADEKQLEILQDKANFRSF-KPKPFNMREFY  158
ratASIC3       120  PAEHAA--------YLRALGQPPAPPGFMPSPTFDMAQLY  151
chickenASIC1a  121  NRYEIPDTQTADEKQLEILQDKANFRNF-KPKPFNMLEFY  159
Consensus_aa:       s.bchs........bLchL.p.ss..sF..s.sFsM.phY
Consensus_ss:       hh          hhhhhhhhhh             hhhhh

Conservation:        9999    999 9  99  9  9 9 9 999 9 99999
ratASIC1a      159  DRAGHDIRDMLLSCHFRGEACSAEDFKVVFTRYGKCYTFN  198
ratASIC3       152  ARAGHSLEDMLLDCRYRGQPCGPENFTVIFTRMGQCYTFN  191
chickenASIC1a  160  DRAGHDIREMLLSCFFRGEQCSPEDFKVVFTRYGKCYTFN  199
Consensus_aa:       sRAGHslc-MLLsC.@RGp.CtsEsFpVlFTRhGpCYTFN
Consensus_ss:       hhh   hhhhhheeee  eee hhheeeee     eeee

Conservation:        9  9   9 9 999 999999999 99 9999 9    9
ratASIC1a      199  SGQDGRPRLKTMKGGTGNGLEIMLDIQQDEYLPVWGETDE  238
ratASIC3       192  SGAHGAELLTTPKGGAGNGLEIMLDVQQEEYLPIWKDMEE  231
chickenASIC1a  200  AGQDGKPRLITMKGGTGNGLEIMLDIQQDEYLPVWGETDE  239
Consensus_aa:       tG.cG...L.T.KGGhGNGLEIMLDlQQ-EYLPlW.-h-E
Consensus_ss:              eeee       eeeeeeee hhh
```

```
Conservation:             9 99 99 9999999999 9999999 999 9999999 9
ratASIC1a          239    TSFEAGIKVQIHSQDEPPFIDQLGFGVAPGFQTFVSCQEQ   278
ratASIC3           232    TPFEVGIRVQIHSQDEPPAIDQLGFGAAPGHQTFVSCQQQ   271
chickenASIC1a      240    TSFEAGIKVQIHSQDEPPLIDQLGFGVAPGFQTFVSCQEQ   279
Consensus_aa:             TsFEhGI+VQIHSQDEPPhIDQLGFGhAPG@QTFVSCQpQ
Consensus_ss:                   eeeeee           eeee   eeeeeeeeeh

Conservation:              9  99 999 9     9    9
ratASIC1a          279    RLIYLPSPWGTCNAVTMDSDFFD----------------S   302
ratASIC3           272    QLSFLPPPWGDCNTASLDPDDFDPEPSDPLGSPRPRPSPP   311
chickenASIC1a      280    RLIYLPPPWGDCKATTGDSEFYD----------------T   303
Consensus_aa:             pL.@LPsPWGsCphho.Ds-.@D................s
Consensus_ss:             heee

Conservation:             99   99  99 99    9 999 9999  9 9 9 999
ratASIC1a          303    YSITACRIDCETRYLVENCNCRMVHMPGDAPYCTPEQYKE   342
ratASIC3           312    YSLIGCRLACESRYVARKCGCRMMHMPGNSPVCSPQQYKD   351
chickenASIC1a      304    YSITACRIDCETRYLVENCNCRMVHMPGDAPYCTPEQYKE   343
Consensus_aa:             YSlhtCRlsCEoRYlhcpCsCRMhHMPGstPhCoPpQYK-
Consensus_ss:             hhhhhhhhhhhhhhhhhh            ee  hhhhhh

Conservation:             99 9999    99   999  99   999 999999 999
ratASIC1a          343    CADPALDFLVEKDQEYCVCEMPCNLTRYGKELSMVKIPSK   382
ratASIC3           352    CASPALDAMLRKD--TCVCPNPCATTRYAKELSMVRIPSR   389
chickenASIC1a      344    CADPALDFLVEKDNEYCVCEMPCNVTRYGKELSMVKIPSK   383
Consensus_aa:             CAsPALDhhlcKD..hCVC..PCshTRYtKELSMV+IPS+
Consensus_ss:             hhhhhhhhhh              eeeeeeeeeeee   h

Conservation:             999 999 9 9 99 99 99 99999999 9999  999
ratASIC1a          383    ASAKYLAKKFNKSEQYIGENILVLDIFFEVLNYETIEQKK   422
ratASIC3           390    ASARYLARKYNRSESYITENVLVLDIFFEALNYEAVEQKA   429
chickenASIC1a      384    ASAKYLAKKYNKSEQYIGENILVLDIFFEALNYETIEQKK   423
Consensus_aa:             ASA+YLA+K@N+SEpYIsENlLVLDIFFEhLNYEhlEQK.
Consensus_ss:             hhhhhhhhhh          eeeeeeee    eeeeeeee

Conservation:             999   9999999999999999 99 99  99  99   9
ratASIC1a          423    AYEIAGLLGDIGGQMGLFIGASILTVLELFDYAYEVIKHR   462
ratASIC3           430    AYEVSELLGDIGGQMGLFIGASLLTILEILDYLCEVFQDR   469
chickenASIC1a      424    AYEVAGLLGDIGGQMGLFIGASILTVLELFDYAYEVIKHR   463
Consensus_aa:             AYElt.LLGDIGGQMGLFIGASlLTlLElhDYhhEVhpcR
Consensus_ss:                hhhhhhhhhhhhhhhhhhhhhhhhhhhhhhhhhhhhh
```

```
Conservation:                                9 9  99   9
ratASIC1a      463  LCRRGKCQKEAKRSSADKGVALSLDDVKRHNPCESLRGHP  502
ratASIC3       470  VLGYFWNRRSAQKRSGNTLLQEELNGHRTHVPHLSLGPRP  509
chickenASIC1a  464  LCRRGKCRKNHKRNNTDKGVALSMDDVKRHNPCESLRGHP  503
Consensus_aa:       lh...bsp+php+psssp.l.bphssh+pHsPhbSL.s+P
Consensus_ss:       hhhhhhhhh  hh

Conservation:                     9          9
ratASIC1a      503  AGMT--YAANILPHHPARGTFEDFTC------  526
ratASIC3       510  PTTPCAVTKTLSASHRT--------CYLVTRL  533
chickenASIC1a  504  AGMT--YAANILPHHPARGTFEDFTC------  527
Consensus_aa:       sshs..hh.sl.spH.h........C......
Consensus_ss:                                eeeee
```

Supplementary Table 1. pH_{50} and Hill coefficient of ASIC1a WT and histidine mutants in the extracellular domain of ASIC1a during activation.

	**pH_{50}**	**Hill coefficient**	**Sample size**	**Statistics**
**ASIC1a WT**	6.3 ± 0.05	2.7 ± 0.2	7	
**ASIC1a-H72A**	6.2 ± 0.05	2.8 ± 0.2	5	$p > 0.05$
**ASIC1a-H73A**	6.2 ± 0.06	2.6 ± 0.1	5	$p > 0.05$
**ASIC1a-H110A**	6.2 ± 0.09	2.8 ± 0.2	5	$p > 0.05$
**ASIC1a-H163A**	6.2 ± 0.05	2.7 ± 0.1	5	$p > 0.05$
**ASIC1a-H173A**	6.3 ± 0.04	2.7 ± 0.1	5	$p > 0.05$
**ASIC1a-H250A**	6.3 ± 0.06	2.6 ± 0.1	5	$p > 0.05$
**ASIC1a-H327A**	6.2 ± 0.07	2.8 ± 0.2	5	$p > 0.05$

pH_{50} and Hill value of each mutant were compared with ASIC1a WT. Data were presented as mean ± SEM.

Supplementary Table 2. pH_{50} and Hill coefficient of ASIC3 WT and histidine mutants in the extracellular domain of ASIC3 during activation of the channel

	**pH_{50}**	**Hill coefficient**	**Sample size**	**Statistics**
**ASIC3 WT**	6.8 ± 0.03	1.4 ± 0.1	6	
**ASIC3-H72A**	6.8 ± 0.02	1.3 ± 0.1	5	$p > 0.05$
**ASIC3-H73A**	6.8 ± 0.03	1.4 ± 0.1	5	$p > 0.05$
**ASIC3-H83A**	6.8 ± 0.03	1.3 ± 0.1	5	$p > 0.05$
**ASIC3-H109A**	6.9 ± 0.02	1.3 ± 0.1	5	$p > 0.05$
**ASIC3-H123A**	6.8 ± 0.03	1.4 ± 0.1	5	$p > 0.05$
**ASIC3-H156A**	6.8 ± 0.02	1.4 ± 0.1	5	$p > 0.05$
**ASIC3-H195A**	6.8 ± 0.03	1.4 ± 0.1	5	$p > 0.05$
**ASIC3-H243A**	6.9 ± 0.02	1.3 ± 0.1	5	$p > 0.05$
**ASIC3-H262A**	6.8 ± 0.03	1.4 ± 0.1	5	$p > 0.05$
**ASIC3-H336A**	6.8 ± 0.03	1.3 ± 0.1	5	$p > 0.05$

pH_{50} and Hill value of each mutant compared with ASIC1a WT. Data were presented as mean ± SEM.
